# Femtosecond X-ray emission study of the spin cross-over dynamics in haem proteins

**DOI:** 10.1038/s41467-020-17923-w

**Published:** 2020-08-18

**Authors:** Dominik Kinschel, Camila Bacellar, Oliviero Cannelli, Boris Sorokin, Tetsuo Katayama, Giulia F. Mancini, Jérémy R. Rouxel, Yuki Obara, Junichi Nishitani, Hironori Ito, Terumasa Ito, Naoya Kurahashi, Chika Higashimura, Shotaro Kudo, Theo Keane, Frederico A. Lima, Wojciech Gawelda, Peter Zalden, Sebastian Schulz, James M. Budarz, Dmitry Khakhulin, Andreas Galler, Christian Bressler, Christopher J. Milne, Thomas Penfold, Makina Yabashi, Toshinori Suzuki, Kazuhiko Misawa, Majed Chergui

**Affiliations:** 1 Laboratoire de Spectroscopie Ultrarapide (LSU) and Lausanne Centre for Ultrafast Science (LACUS), Ecole Polytechnique Fédérale de Lausanne (EPFL), 1015 Lausanne, Switzerland; 2 Japan Synchrotron Radiation Research Institute (JASRI), 1-1-1, Kouto, Sayo-cho Sayo-gun, Hyogo 679-5198 Japan; 3 Tokyo University of Agriculture and Technology (TUAT), 2-24-16 Naka-cho, Koganei, Tokyo 184-8588 Japan; 4 Department of Chemistry, Graduate School of Science, Kyoto University, Kitashirakawa-Oiwakecho, Sakyo-Ku, Kyoto 606-8502 Japan; 5 Department of Materials and Life Sciences, Faculty of Science and Technology, Sofia University, 102-8554 Tokyo, Chiyoda, Kioicho 7-1 Japan; 6 Chemistry-School of Natural and Environmental Science, Newcastle University, Newcastle upon Tyne, NE1 7RU UK; 7 European XFEL, Holzkoppel 4, 22869 Schenefeld, Germany; 8 Faculty of Physics, Adam Mickiewicz University, 61-614 Poznań, Poland; 9 SwissFEL, Paul-Scherrer-Institut (PSI), 5232 Villigen, Switzerland

**Keywords:** Biophysical chemistry, Excited states

## Abstract

In haemoglobin the change from the low-spin (LS) hexacoordinated haem to the high spin (HS, S = 2) pentacoordinated domed deoxy-myoglobin (deoxyMb) form upon ligand detachment from the haem and the reverse process upon ligand binding are what ultimately drives the respiratory function. Here we probe them in the case of Myoglobin-NO (MbNO) using element- and spin-sensitive femtosecond Fe K_α_ and K_β_ X-ray emission spectroscopy at an X-ray free-electron laser (FEL). We find that the change from the LS (S = 1/2) MbNO to the HS haem occurs in ~800 fs, and that it proceeds via an intermediate (S = 1) spin state. We also show that upon NO recombination, the return to the planar MbNO ground state is an electronic relaxation from HS to LS taking place in ~30 ps. Thus, the entire ligand dissociation-recombination cycle in MbNO is a spin cross-over followed by a reverse spin cross-over process.

## Abstract (web summary)

The change from low-spin hexacoordinated to high-spin pentacoordinated domed form in heam upon ligand detachment and the reverse process underlie the respiratory function. The authors, using femtosecond time-resolved X-ray emission spectroscopy, capture the transient states connecting the two forms in myoglobin-NO upon NO photoinduced detachment.

## Introduction

**Fig. 1:**
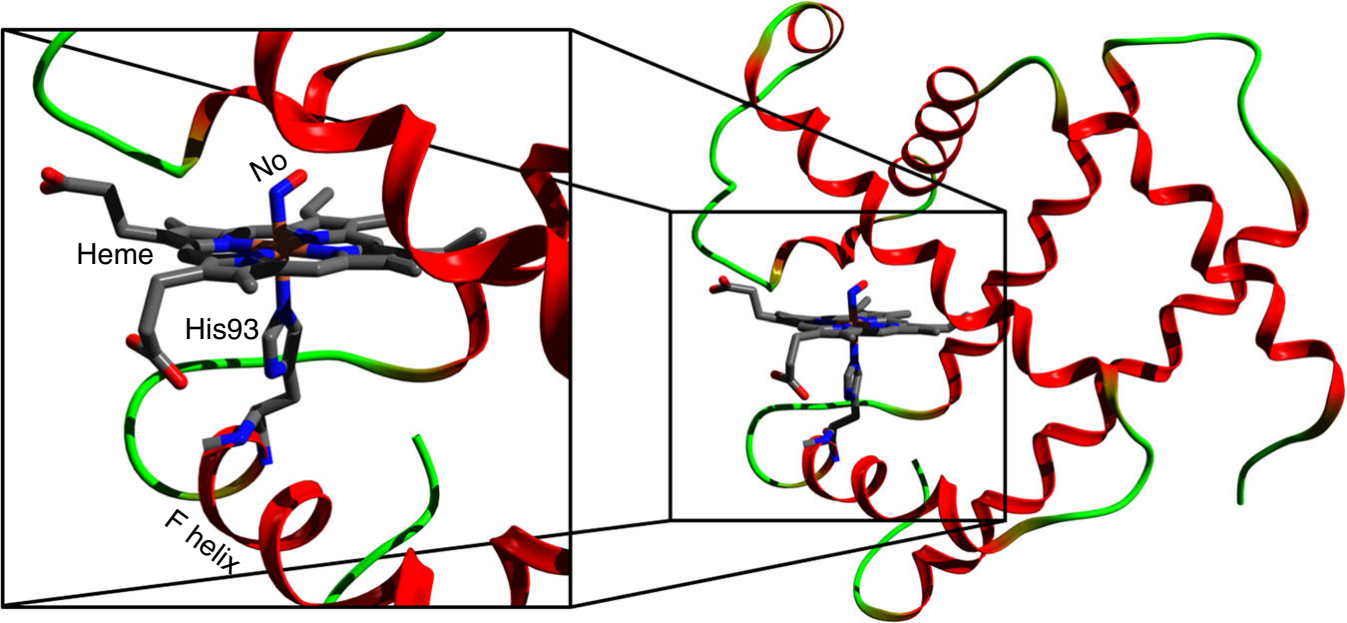
Crystal structure of the nitrosyl-myoglobin (MbNO) (PDB entry: 10.2210/pdb1hjt/pdb). The haem is represented as sticks (Fe [orange], C [grey], N [blue], O [red]). The zoom (left) shows the distal NO ligand and proximal histidine 93 (His93), which links the Fe atom of the haem porphyrin to the F helix.

Small ligand molecules (O_2_, CO, NO, CN and H_2_O) are the receptors that bind to and activate haem proteins, such as myoglobin (Mb) and haemoglobin (Hb). Their active site (Fig. 1) is a porphyrin with an Iron ion (that can be either ferric-Fe^3+^ or ferrous-Fe^2+^) at its centre to which the ligands bind on the distal side. The haem itself is linked to the F helix of the protein via a histidine (His) amino-acid bound to the Fe ion on the proximal side of the porphyrin. The ferrous forms of Mb and Hb play an essential role in respiration via the transport and storage of oxygen in mammals. In the ligated form, the hexacoordinated Fe^2+^ ion of the haem is in a low-spin (LS) planar singlet (MbCO) or doublet state (MbNO). Upon release of the ligand from the Fe^2+^ ion, the haem switches to a high-spin (HS) quintet state with the pentacoordinated Fe in an out-of-plane, so-called domed, deoxyMb configuration (Supplementary Fig. 1). The shuttling between the LS planar to HS-domed spin cross-over (SCO) process defines the “transition state”, which governs the allosteric transitions in haem proteins (including O_2_ carriers and NO-sensor and CO-sensor)^1,2^. In particular it is the first event of the respiratory function in which, after release of the distal ligand, the Fe atoms moves out of the porphyrin plane, pushing the proximal His linked to the F helix and displacing the latter (Fig. 1). This is considered the step by which the monomer units of Hb transfer information, ultimately leading to the transition from the so-called “relaxed” to “tense” state at later times^3^.

It has long been known that ligand detachment in ferrous Mb’s can be induced by visible–ultraviolet (UV) excitation of π−π* transitions of the haem^4^. This has been exploited to mimic the release of the distal ligand from the Fe centre, and monitor its recombination to the haem. With the advent of ultrafast spectroscopy, haem proteins were among the first systems ever to be studied, specifically with the aim of monitoring these processes in “real-time” using transient absorption (TA) spectroscopy from the visible–UV (sensitive to the porphyrin π−π* transitions)^5–9^ to the Infrared (IR) (sensitive to the ligand stretch and the porphyrin vibrational modes)^10,11^, as well as by time-resolved resonance Raman spectroscopy (sensitive to the Fe–His bond)^12,13^. These studies concluded that photodissociation of the diatomic ligands and doming are prompt and simultaneous events, typically in <50–100 femtoseconds (fs)^14^. The actual formation of the ground state HS deoxyMb form has been described by two main and non-mutually excluding models: (a) a vibrationally hot ground-state is formed promptly after excitation, in which cooling ensues^8,15,16^ and (b) the process is a cascade through intermediate electronic excited states^6,17^.

**Fig. 2:**
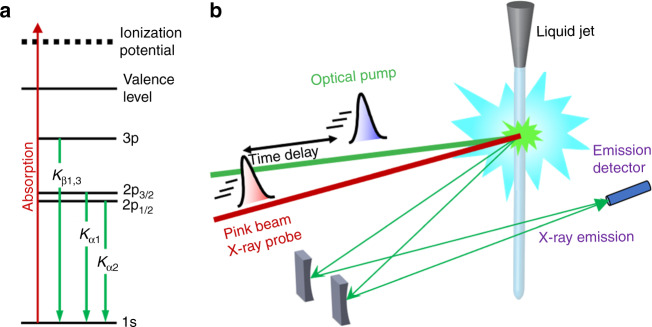
Experimental scheme. **a** Energy level diagram showing the origin of the K_α_ and K_β_ fluorescence after creation of a hole in the 1*s* (K) shell. The K_α1_ and K_α2_ lines originate from the splitting of the 2*p* orbital (2*p*_1/2_ and 2*p*_3/2_), whereas for K_β_ these lines are degenerate, resulting in the line labelled K_β1,3_. **b** Experimental setup for the time-resolved X-ray emission spectroscopy measurements at the XFEL. The green line represents the optical (533 nm) pump pulse used to excite the sample and the red line represents the X-ray (centred at 8.168 keV) probe pulse at varying time delays (black arrow). The pulses are overlapped and intercept the sample at the green interaction region. A von Hamos geometry was used for these measurements.

Recently, the occurrence of prompt doming was questioned in a fs hard X-ray absorption spectroscopy (XAS) study of photoexcited MbCO^18^. Time-resolved XAS is an ideal element-selective probe of the electronic and geometric structure changes of metal-containing molecular complexes^19^. Using a monochromatic probe at 7.123 keV (tuned to the Fe K-edge), Levantino et al. ^18^ observed a first <50–70 fs event, which they attributed to CO photolysis from the LS (*S* = 0) MbCO and partial SCO to a higher spin state, followed by a further 300–400 fs event, attributed to the passage to the HS (*S* = 2) state of deoxyMb. This picture was supported by quantum wave packet dynamics simulations^20^, which accounted for coherent nuclear and electronic motions. However, Levantino et al.’s^18^ use of a monochromatic probe hindered a spectroscopic identification of the intermediate and final states, let alone of their spin. In recent years, non-resonant X-ray emission spectroscopy (XES) has emerged as a valuable tool to identify spin states of transition metal complexes (TMC)^21^. The K_β1,3_ and $K_\beta^\prime$ lines (3*p* → 1*s* emission, Fig. 2a) have been used as markers of the spin state (number of unpaired 3*d* electrons) of TMC’s via the 3*p*–3*d* exchange interactions and fs transient K_β_ XES was successfully implemented to monitor the SCO dynamics in [Fe(bpy)_3_]^2+^^22^, as well as to identify the HS deoxy haem product of photoexcited ferrous cytochrome c after dissociation of its distal methionine ligand^23^. In the case of 2*p*-orbitals, the *p*–*d* exchange is weaker but K_α_ XES (2*p* → 1*s* transitions, Fig. 2a) can also be used as a marker of spin state via the linear dependence of the full-width at half-maximum (FWHM) of the K_α1_ line as a function of the number of unpaired metal 3*d* electrons^24^.

In this contribution, we focus on the case of nitrosylmyoglobin (MbNO). The role of nitric oxide (NO) in physiological processes in humans was discovered in the 1990s^25^. In its interaction with haem systems, the NO molecule plays an important role in several physiological effects and biological functions, such as neurotransmission^26^, regulation of vasodilatation^27^, platelet aggregation^28^, and immune response^29^. Furthermore, studies of NO release from and binding to the haem provide mechanistic information of relevance for other ligands active in respiration, blood pressure regulation, nitrogen fixation, and NO oxidation. In this respect, several time-resolved studies using visible–UV and IR probes have been carried out on MbNO over the past three decades. Just as for MbCO, NO photodissociation from the haem has been reported to be prompt (<100 fs) with a quantum yield of ~55%^7^, and recovery of the system occurs on typical timescales of 1–3, 5–20, 110–290 ps, and a weak (~10–20%) ns component^7,11,13,30–37^. The shortest recovery time was attributed to cooling within what was considered, the promptly formed hot ground state of deoxyMb^8^, while the next two decay times were attributed to geminate recombination to the latter of, respectively, NO ligands from the distal pocket close to the haem and NO ligands from the more distant Xe4 pocket. Finally, the nanosecond (ns) component is due to non-geminate recombination of a small fraction of NO ligands that have escaped the protein. Contrary to other ligands (CO in particular), which can bind to a planar haem, the unpaired NO electron allows its binding to the domed HS deoxyMb^36^, and this was recently supported by a time-resolved Fe K-edge X-ray near edge structure (XANES) study with 70 ps resolution^37^. The binding of NO to a domed deoxyMb (hereafter designated as deoxyMb–NO) had previously been predicted by theoretical calculations on model haems^38^, which argued that the return to the planar form is a reverse SCO from HS deoxyMb–NO to LS planar MbNO. Recent ultrafast resonance Raman studies^13^ monitoring the Fe–His bond (Fig. 1) determined that the domed-to-planar transition in MbNO takes place in ~30 ps and argued that the process is determined by  constraints exerted by the protein structure on the haem cofactor (Fig. 1). Since the entire ligand dissociation–recombination cycle occurs within ~250 ps, and is much shorter than the commonly studied MbCO, this implies that it can be monitored within the same ultrafast spectroscopic experiment.

In order to identify the mechanisms leading to the formation of HS deoxyMb in myoglobins and its time scale, and the specific case of the reverse deoxyMb to MbNO transition, here we use fs Fe K_α_ and K_β_ non-resonant XES with a von Hamos spectrometer (Fig. 2b) at the SPring-8 angstrom compact free electron laser (SACLA)^39,40^. We record laser-off and laser-on XES spectra (whose difference yields the transient XES) as a function of time delay with respect to the pump pulse. We find that upon 533 nm photoexcitation of MbNO into the Q-band (Supplementary Fig. 2), the SCO from the ground state LS (*S* = 1/2) MbNO^41^ to the HS (*S* = 2) deoxyMb occurs in ~800 fs and involves the passage via an intermediate S = 1 spin state, while the return from the HS deoxyMb–NO to the LS MbNO is a reverse SCO that takes place in ~30 ps. We thus establish the details of domed HS deoxyMb formation, which we argue is of general validity to all Mb’s, and we identify the return to the initial MbNO ground state also as a HS–LS relaxation. The observation of the *S* = 1 state is supported by density functional theory (DFT) calculations of the K_α_ XES. Details of the experimental set-up and procedures and of the theoretical calculations are given in the SI.

## Results and discussion

### X-ray emission transients

In previous ultrafast^22,23^ or quasi-static^42^ K_β_ XES studies, the assignment of spin states relied on the comparison with reference spectra of model compounds. Supplementary Fig. 3 shows the steady-state K_β_ emission spectra of hexacoordinated MbNO and pentacoordinated deoxyMb and compares them with those of reference Fe-based molecular complexes in Supplementary Fig. 3b^22^, and reference Fe(II) LS and HS porphyrin compounds^42^. While the trends are similar as a function of increasing the spin in all three panels, with the intensity increase of the K_β’_ line around 7045 eV and a blue shift and intensity decrease of the K_β1,3_ line near 7058 eV, some differences also show up in that the blue shift of the K_β1,3_ line in Supplementary Fig. 3a and its energy splitting with respect to the K_β’_ line are smaller than in Supplementary Fig. 3b and c. These differences may point to a covalency change^43–46^ in addition to the spin change, as will be discussed later. Supplementary Fig. 4a shows the steady-state K_α_ lines of MbNO and deoxyMb. There is an intensity decrease and slight blue shift and broadening of the K_α1_ line with increasing spin, as reported for other compounds^24^, while the K_α2_ line undergoes an intensity decrease and a slight broadening. Because K_α_ XES spectra of reference compounds are lacking, we resorted to DFT to simulate them (see Supplementary Note 4), as discussed later. In summary, the above XES spectra for the Mb LS hexacoordinated and the HS pentacoordinated forms provide the two extreme cases before and after photodissociation of the ligand, expected in time-resolved experiments, which allow us to identify the intermediate steps.

**Fig. 3:**
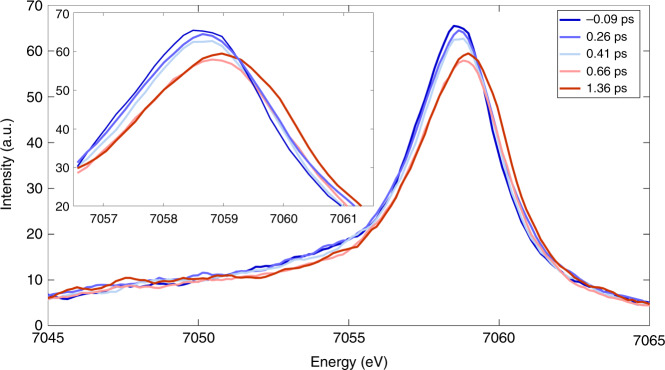
K_β_ XES spectra of MbNO at various time delays after visible photoexcitation. Laser-off (−0.09 ps) and Laser-on K_β_ XES spectra of MbNO at different time delays between 0.026 and 1.36 ps (from blue to red) showing a blue shift of the K_β1,3_ line and an intensity decrease. The inset zooms into the region of the maximum of $K_{\beta _{1,3}}{\mathrm{{XES}}}$ line at different time delays between −0.09 and 1.36 ps (from blue to red) showing peak shifts smaller than the energy resolution (~0.5 eV) of our experiment.

The laser-off and laser-on K_β_ spectra are shown in Fig. 3 for time delays up to ~1.4 ps, while the inset zooms into the region of the maximum of the K_β1,3_ line. These spectra show an intensity decrease and blue shift of the latter, an intensity decrease in the 7050–7055 eV region and a slight increase of the K_β´_ line in the 7045–7050 eV region, all of which reflect the trends predicted for a change from LS to HS Mb (Supplementary Fig. 3a) and qualitatively agree with reference Fe(II) complexes (Supplementary Fig. 3b and c)^22^. Laser-on spectra at later times are shown in Supplementary Fig. 5, and they exhibit a return to the laser-off spectra on tens to hundreds of ps. Further insight into these changes is obtained from the normalized transient K_β_ XES spectra (excited minus ground state signal) shown in Supplementary Fig. 6a for time delays up to 1.36 ps, and Supplementary Fig. 6b for time delays up to 100 ps. These transients exhibit a derivative-like shape near 7058 keV, a negative signal that extends down to 7050 eV and a positive one below in the region of the K_β´_ line. Beyond ~1 ps, the normalized transient spectra no longer change but before, the 7050–7057 eV region shows a gradual increase of negative amplitude, while the peak near 7060–7062 eV is most blue shifted at 0.26 ps, and then shifts slightly to the red and stabilizes at 7060 eV for times >1 ps. The evolution of the 7050–7057 eV region qualitatively reproduces the static difference spectra of reference Fe-complexes of different spins (Supplementary Figs. 7 and 19), that revealed the transient population of a triplet state in the photoinduced SCO of [Fe(bpy)_3_]^2+^^22^. From Fig. 3 and Supplementary Fig. 6a, we conclude that reaching the final HS deoxyMb state requires ~1 ps and proceeds via an intermediate state.

**Fig. 4:**
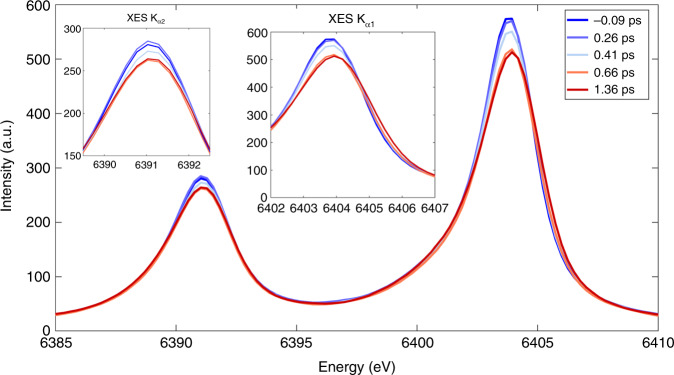
K_α_ XES spectra of MbNO at various time delays after visible photoexcitation. Laser-on (−0.09 ps) and laser-om $K_\alpha$ XES spectra of MbNO at different time delays between 0.26 and 1.36 ps (from blue to red) showing an intensity decrease for K_α2_ over the first 1.2 ps after excitation and an intensity decrease and change in peak width for K_α1_. Also, the visibility of changes in peak width (*σ*) smaller than the energy resolution (~0.6 eV) can be well observed. The insets zoom into the peaks of the K_α2_ and K_α1_ laser-on (pumped) XES spectra of MbNO at different time delays between −0.09 and 1.36 ps (from blue to red) showing an intensity decrease for K_α2_ over the first 1.2 ps after excitation and an intensity decrease and change in peak width for K_α1_. The changes in peak width (*σ*) smaller than the energy resolution (~0.6 eV) can be well observed.

Figure 4 shows the laser-off and laser-on K_α1_ and K_α2_ XES lines at different time delays, while the insets zoom into the region of their maxima. One notices a clear gradual weakening and a slight broadening (and for K_α1_, a slight blue shift) of the bands within the first ps. The trends in energy, width and intensity of these lines are consistent with the differences we observe between MbNO and deoxyMb (Supplementary Fig. 4), but in Fig. 4, the evolution is gradual and on a similar time scale as for the K_β_ lines. Supplementary Fig. 8 shows the laser-on spectra at later times, and just as in Supplementary Fig. 6, they exhibit a return to the laser-off spectra on tens to hundreds of ps. In order to highlight the changes, Supplementary Fig. 9 shows the experimental K_α_ XES transients at 0.26 and 1.36 ps time delay, while those at intermediate and at later times are shown in Supplementary Fig. 10. The normalized transient line shapes reflect the broadening, shift and intensity changes of the asymmetric emission lines, which do not change beyond ~1 ps, by which time the system is in the HS state, as confirmed by the difference of the experimental steady-state spectra deoxyMb minus MbNO (Supplementary Fig. 9b). In summary, both the K_α_ and K_β_ XES transients point to an intermediate state being populated prior to the HS deoxyMb state that is fully formed by ~1 ps.

### X-ray emission kinetics

The signal around 7053 eV in Fig. 3 and Supplementary Fig. 6 best differentiates the intermediate state from the initial excited LUMO state to the quintet state. As already mentioned, this is where the signature of an intermediate spin state was most pronounced in the previous study on [Fe(bpy)_3_]^2+^^22^. By plotting its amplitude as a function of time for the transients available up to 3.6 ps, we can get an estimate of the lifetime of the intermediate state, using a three-level kinetic model (Supplementary Note 10 and Supplementary Fig. 11), assuming 100% Q-state population at *t* = 0 that decays to the triplet state in ~100 fs, as concluded from ultrafast fluorescence up-conversion studies^47^. The fit yields a lifetime of 500 ± 250 fs for the intermediate state (Supplementary Figs. 12 and 13).

**Fig. 5:**
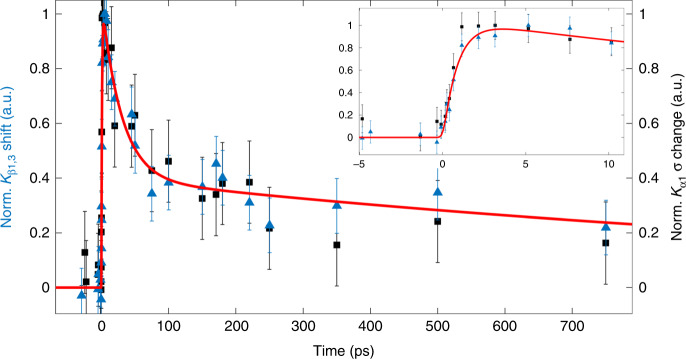
Kinetic traces. Temporal evolution of the relative $K_{\beta _{1,3}}$ shift for MbNO (blue triangles, centroid obtained from Gaussian fit, max shift is ~0.45 eV, see Supplementary Note 6) and of the $K_{\alpha _1}$ peak width changes for MbNO (black squares, normalized *σ* obtained from a Gaussian fit, maximum change in *σ* is ~0.3). The red line is a fit of the experimental data using a function consisting of a rising component and a biexponential decay. The former corresponds to a risetime of 800 ± 150 fs (see Supplementary Note 6 and Supplementary Fig. 18), significantly longer than our cross-correlation (~150 fs), while the decay components have time constants (pre-exponential factors) of *τ*_1_ = 30 ± 9 ps (*A*_1_ = 0.6) and *τ*_2_ = 1.5 ± 0.9 ns (*A*_2_ = 0.4). The inset shows the temporal evolution during the first 10 ps of both signals. The error bars represent the 1-sigma uncertainty of the Gaussian fit for the spectra at each time-point. Details are given in Supplementary Note 6 and Supplementary Fig. 18.

Further details into the ultrafast kinetics comes from the peak position of the laser-on $K_{\beta _{1,3}}$ line (Fig. 3 and Supplementary Fig. 5) and the full width at half maximum (FWHM) of the laser-on K_α1_ line (Fig. 4 and Supplementary Fig. 8), both of which are sensitive to spin. During the first ps, these spectra reflect a mixture of doublet, short-lived intermediate state and of the final quintet state, before the entire excited population settles in the latter. The K_β1,3_ peak energy shift and the FWHM of the K_α1_ line are plotted in Fig. 5 for times up to 750 ps, while the inset zooms into the first 10 ps. The red trace is a fit of the experimental data using a function consisting of a rising component and a biexponential decay. The former corresponds to a risetime of 800 ± 150 fs (see Supplementary Note 6), significantly longer than our cross-correlation (~150 fs), while the decay components have time constants (pre-exponential factors) of *τ*_1_ = 30 ± 9 ps (*A*_1_ = 0.6) and *τ*_2_ = 1.5 ± 0.9 ns (*A*_2_ = 0.4). Interestingly, while the UV–visible and IR TA results are characterized by a prompt risetime and decay times of 5–20, 110–290 ps, and a weak ns component^7,11,13,30–37^, the present XES observables exhibit different kinetics with a rise of ~800 fs and decays of ~30 ps and ns’s. As mentioned previously, the ~30 ps decay had been reported in the time-resolved resonance Raman studies by Kruglik et al.^13^, who attributed it to the relaxation from the domed HS deoxyMb–NO species to the LS planar MbNO. Given the spin sensitivity of XES our observation of the ~30 ps decay is fully consistent with this interpretation as it reflects the return of the system to the LS ground state. Since the trigger to this process is the binding of the NO ligand to deoxyMb, in principle the geminate recombination (in 5–20 and 110–250 ps) and the non-geminate recombination (ns component) should feed the 30 ps component. In this scenario, the 5–20 ps component would appear as a rise time, while the 110–250 ps and the ns components would show up as decay because they are rate-determining to the 30 ps component. However, due to the scatter of data points at early times in Fig. 5, we refrained from extracting such times from the fits, preferring the above phenomenological function, which captures the important features using a single rise time and a biexponential decay. In this respect, the relatively large pre-exponential factor of the ns decay component, compared to previous reports (see ref. ^37^ and references therein), most likely reflects the fact that it includes both geminate (110–250 ps) and non-geminate (ns) recombination of NO ligands.

Coming back to the short time dynamics, we have yet to identify the intermediate state between the initial MbNO excited state and the HS deoxyMb. The changes occurring at early times (Fig. 3 and Supplementary Fig. 5) reflect the transient population of an intermediate, most likely an *S* = 1 spin state, if we parallel the present results with those on photoexcited [Fe(bpy)_3_]^2+^^22^. We further support the assignment of the intermediate state as having *S* = 1 using DFT calculations, presented in Supplementary Note 4 and Supplementary Figs. 14, 15 and 20. However, in addition to its spin sensitivity, K_β_ XES is also sensitive to metal–ligand covalency^43–46^, which represents the charge donation from the ligands to the metal and therefore, it has a significant influence on the nature of metal–ligand bonds. The degree of covalency is connected via the effective number of unpaired 3*d* electrons to the magnitude of the exchange splitting. In K_β_ XES, it manifests itself in, among others, the splitting between the K_β1,3_ and K_β’_ lines. Thus, covalency could also affect the transient spectra as a result of dissociating the NO ligand and of the relaxation to the HS state, which involves an elongation of the Fe–Np bonds (Np are the pyrrole nitrogens). We hold it however unlikely that covalency effects affect our results: (a) Given the recombination times of NO to deoxyMb (5–20 and 110–290 ps), it seems surprising that the K_β_ transient XES no longer changes beyond 1 ps (Supplementary Fig. 5). This is consistent with the K_β_ XES predominantly reporting on the spin state of the metal, i.e. since it does not distinguish between the HS deoxyMb and HS deoxyMb–NO species; (b) Also, the invariance of spectra in the relaxation from NO–deoxyMb to planar MbNO in ~30 ps is to note, as this corresponds to a contraction of the Fe–Np bonds; (c) Thus, neither NO rebinding nor the ensuing relaxation from domed to planar seem to affect the K_β_ XES. A fortiori, we would also exclude a contribution of covalency at early times (<1 ps), since the reverse processes occur therein (ligand detachment, Fe–Np bond elongation); (d) It should also be noted that the K_β_ XES changes reported in the case of photoexcited [Fe(bpy)_3_]^2+^, where a significant bond elongation occurs^48^ towards the HS state, were fully interpreted in terms of transient population via an intermediate spin state based on reference spectra^22^; (f) Last but not least, covalency effects have not been reported on K_α_ XES and the very close correspondence between the temporal evolutions of the K_α_ and K_β_ XES (Fig. 5) suggests that signature of covalency is negligible in the latter.

**Fig. 6:**
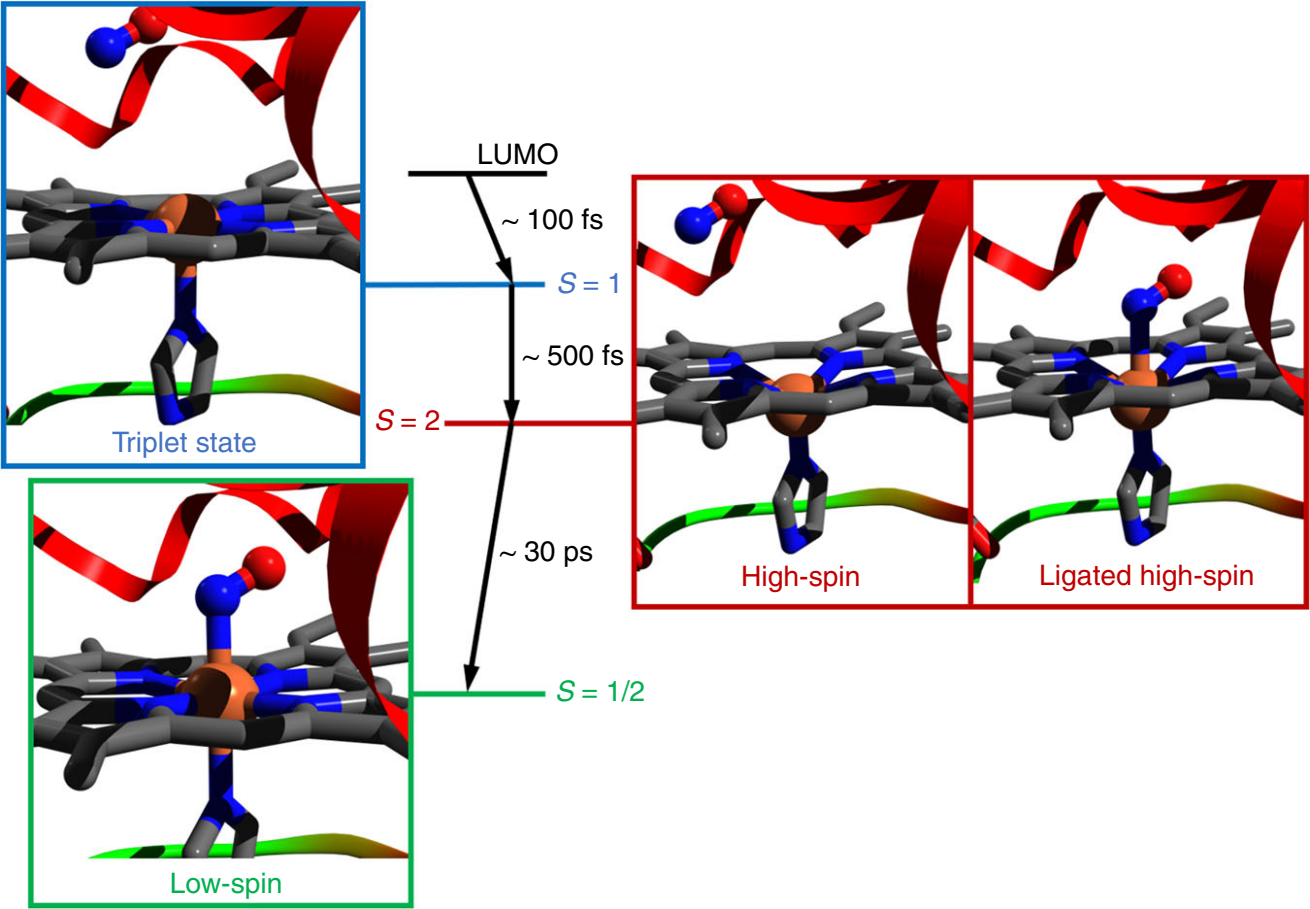
Scheme of the spin cross-over (SCO) and reverse SCO characterizing the photocycle of MbNO. The low-spin ground state MbNO (*S* = 1/2) haem (lower left panel) undergoes prompt dissociation of NO upon π−π* excitation. The porphyrin Q-state decays to a triplet state (*S* = 1, upper left panel) of the pentacoordinated deoxyMb haem in <100 fs^47^. Further relaxation to the quintet state of the pentacoordinated haem occurs in ~500 ± 250 fs (right “High-Spin” panel). The entire process (NO dissociation and relaxation from the Q-state to the high-spin quintet state) occurs in ~800 fs. Upon recombination of NO to deoxyMb leading to a hexacoordinated HS-domed haem (right “ligated high-spin” panel), relaxation back to the LS planar ground state occurs in ~30 ps. The initial low spin structure is PDB entry 2FRJ (Fe = orange, N = blue, O = red and C = grey), the sizes of the Fe atom, the NO molecule and the doming are exaggerated in order to highlight the key changes in the photocycle. The actual structural differences between MbNO and deoxyMb are shown in Supplementary Fig. 1.

To summarize our results, Fig. 6 shows the complete photocycle of MbNO: Upon photoexcitation into the Q-bands, prompt (<100 fs) photodissociation of NO occurs leaving an excited pentacoordinated haem that undergoes doming by first relaxing to an intermediate spin state (*S* = 1), followed by a second relaxation step to the HS *S* = 2 state forming deoxyMb. The entire cascade occurs in ~800 fs. The relaxation back from the domed HS haem to the planar ground state occurs in ~30 ps upon NO recombination. The latter takes place over several time scales due to geminate (5–20, 110–200 ps) and non-geminate recombination (ns’s). These results also show that the relaxation from domed NO–deoxyMb to the planar MbNO is a genuine HS to LS relaxation, as theoretically predicted by Franzen^38^, although constraints due to the F-helix (Fig. 1)^13^ cannot be fully excluded. Thus, the entire cycle of detachment and rebinding of NO to deoxyMb is accounted for by a SCO followed by a reverse SCO (Fig. 6). There are many parameters that affect the rates of forward-SCO and back-SCO but in the context of our interpretation, it is clear that the former is a stepwise process by jumps of Δ*S* = 1, which are ultrafast in metal complexes^49^, while the reverse SCO is a Δ*S* = 2 process occurring on a much slower time scale.

The above results have several implications on the description of the energy flow in haem proteins: (a) upon ligand detachment, the formation of the fully domed deoxyMb requires ~800 fs, as it proceeds via an intermediate triplet state, which decays in 500 ± 250 fs; (b) The ligand detachment is here mimicked by a photodissociation, which is prompt (<50 fs), implying that the ensuing dynamics is taking place in the pentacoordinated haem and should therefore be the same for all ferrous haems involved in ligand release. This was already suggested by Ye et al. ^8^ based on UV–visible TA studies, but they concluded that the ensuing evolution within the deoxyMb product is due to relaxation of a vibrationally hot species, while we conclude that it is an electronic relaxation cascade. The debate to determine the initial pathways for ultrafast energy flow in haem proteins, has been going one for over 30 years now, with the interpretation about formation of deoxyMb going from an electronic cascade via spin states^5,6^, to a vibrational energy redistribution^8,16^. Here by using element-specific and spin-specific observables (XES), that are not sensitive to thermal and vibrational effects, we show that the initial scenario of a cascade among spin states is operative. Nevertheless, the cascade is non-radiative and energy gaps between spin states are dissipated in the form of heat to the environment. We believe that this behaviour is not limited to ferrous haems (that undergo distal ligand dissociation) but it also includes ferric haems (that remain hexacoordinated) as suggested by the conclusions of UV–visible studies on metMb^50^ and MbCN^51^. Finally, doming has been suggested to trigger large-scale conformational changes of the protein, the so-called “protein quake”, that opens a channel for ligand escape from the protein^52^. Our results show this is unlikely to be a very fast process, contrary to earlier claims that assumed it to occur on the time scale of the Fe–ligand bond breaking^53^.

The present work identifies the transition from the planar ligated haem involved in ligand release to the domed deoxy form as a SCO, which we believe is identical in all ferrous haem proteins. However, the ensuing events following ligand recombination are specific to the NO ligand and are characterized by a reverse SCO. This work also shows the power of ultrafast XES at unravelling exquisite details of the haem transformations upon ligand release and uptake in haem proteins.

## Methods

### Pump-probe XES measurements and analysis

Femtosecond hard XES measurements were carried out on 4 mM solutions of MbNO in a physiological medium (pH = 7) under inert conditions (He or N_2_ atmosphere) at BL3 at SACLA^40^. The sample solution was delivered through a 0.2 mm-thick round liquid jet and its integrity was continuously monitored by a mobile UV–vis spectrometer. A laser pulse at 533 nm with a ~45 fs FWHM was used to excite the Q-bands of MbNO in a near colinear geometry with the X-ray beam (pink, 8.168 keV). An energy-dispersive X-ray emission spectrometer (von Hamos geometry) and a 2D MPCCD detector are used to record the iron 2*p*–1*s* (K_α_) and 3*p*–1*s* (K_β_) fluorescence spectra. A timing tool was used to measure the X-ray/optical relative arrival time fluctuations on a pulse to pulse basis and sort each shot by its relative arrival time (*σ* = 150 ± 40, Supplementary Fig. 16). For the analysis of the K_β_ transients, we have used reference spectra of Fe-containing compounds from ref. ^22^ (see Supplementary Figs. 3 and 17). For the K_α_ emission, reference spectra are lacking, and we relied on DFT simulated XES spectra for the ground state of MbNO and states of different spins for deoxyMb (see Supplementary Note 4 and Supplementary Fig. 4).

## Supplementary information

Supplementary Information

## Data Availability

Processed data used on the analysis and shown in the figures of this manuscript are available in the Supplementary Information. Raw K_α_ and K_β_ XES data were generated at SACLA and the European XFEL large-scale facilities and are available in the repository: https://figshare.com/articles/dataset/Data_of_Figure_3_-_XES_Kbeta/12636470.
