# Supplementary material for: Femtosecond X-ray emission study of the spin cross-over dynamics in haem proteins
Source: Nat Commun. 2020 Aug 18;11:4145. doi: 10.1038/s41467-020-17923-w (PMC7434878; doi:10.1038/s41467-020-17923-w)
Supplement: Supplementary file 1 — Supplementary Information [file 41467_2020_17923_MOESM1_ESM.docx]

Supplementary Information

**Femtosecond X-ray emission study of the spin cross-over dynamics in heme proteins**

*Kinschel et al.*

# Supplementary Note 1

**Sample preparation:** Lyophilized Myoglobin (equine skeletal muscle, 95-100%, Sigma Aldrich) was dissolved in the sodium phosphate buffer (100 mM, pH 7) to obtain a solution of metMb (4 m). The Sodium phosphate buffer (100 mM, pH 7) was prepared with MiliQ water, sodium phosphate monobasic (BioXtra, 99.0%, Sigma Aldrich) and sodium phosphate dibasic (BioXtra, 99.0%, Sigma Aldrich). It was checked with pH paper and was degassed with N_2_ for at least 24 h before use. DeoxyMb was prepared by adding, under inert conditions, a five-fold molar excess of sodium hydrosulfite (Na_2_S_2_O_4_, 85%, Sigma Aldrich) to a solution of metMb (4 mM). MbNO was prepared by adding, under inert conditions, a fivefold excess of sodium nitrite (NaNO_2_, ReagentPlus, 99.0%, Sigma Aldrich). The sample was kept at all times in a glass bottle with an overpressure of N_2_. The different samples were checked with UV-VIS spectrometry for full conversion and the spectra were compared to literature. At the EXFEL, the liquid jet (100 µm) was kept in a small Kapton chamber with an N_2_ overpressure and at SACLA, the liquid jet (200 µm) was in a chamber with a > 98% He atmosphere.

For the preparation of [Fe(bpy)_3_]^2+^, used as a standard, FeCl_2_ was dissolved in a minimum amount of water and heated up, then 3 equivalents of bipyridine with a slight stoichiometric excess were added and the solution was cooled down and dark red crystals were obtained. These were isolated by filtration and characterized by UV-VIS spectroscopy.

During the measurements, the sample integrity was continuously monitored via its UV-Visible spectrum (Supplementary Figure 2) using a portable spectrometer (AvaSpec-ULS2048). In case of minor changes in the spectrum, the sample was refreshed with a five-fold excess of sodium hydrosulfite and sodium nitrite. After a maximum of 12h of measurements, the sample was replaced by a new one.

# Supplementary Note 2

**Time-resolved X-ray emission spectroscopy (SACLA):** The time-resolved XES measurements were performed at BL3 station^1^ of the SACLA X-ray Free electron laser,^2^ which delivers high-intensity X-ray pulses (pulse energy ~500 µJ) of ~10 fs width at a repetition rate of 30 Hz. The intrinsically broad X-ray photon energy distribution (Δ*E*/*E* ≃ 5*10^−3^) was tuned above the Fe K-edge to 8.168 keV by adjusting the conditions of the accelerator and undulators of SACLA. Non-resonant X-ray emission spectra were recorded in the von Hamos geometry using two Ge(333) crystals at a Bragg angle of θ_B_=63^°^ for Fe K_α_, two Si(531) crystals at θ_B_=73^°^for Fe K_β_ and a multiport charge-coupled device (MPCCD, 30 Hz) for detection. The MPCCD has a pixel size of 50 × 50 *μ*m^2^, an imaging area of 25.6 × 51.2 mm^2^ and a quantum efficiency of ∼0.6 at 7 keV. The probe X-ray (pink beam) was focused on the sample solution by beryllium compound refraction lenses to a focal beam diameter of 8 *μ*m. A small fraction of the X-ray pulse was sampled using a Kapton film to monitor its intensity. The sample-jet was placed in a chamber filled with Helium gas (>98%) to prevent sample oxidation and to reduce X-ray light attenuation in air. The crystals and the MPCCD were placed outside the chamber. The energy resolution, after overlapping contributions from all crystals, was evaluated to be ~0.5 eV for Fe K_β_ and ~0.6 eV for Fe K_α_. As we are detecting shifts of a single peak, the energy resolution is not the limiting factor. This is illustrated in Figure 3 and 4 of the main article, which respectively show the XES K_β_ and K_α_ lines of laser on (pumped) spectra for different early times, where one can clearly measure shifts below the energy resolution of the experiment.

The sample was excited by a 533 nm pulse (14.5 nm spectral width), into the Q-bands of MbNO (Supplementary Figure 2). The pump pulse was generated using an amplified Ti:Sapphire laser system equipped with an optical parametric amplifier (HE-TOPAS, Coherent). The pulse duration of 800 nm light (fundamental) was measured to be ∼25 fs. Figure 2 shows a scheme of the experimental setup for ultrafast XES. The pump-laser light was reduced to 15 Hz using a chopper wheel and focused onto the liquid jet. The cross-correlation of the experiment was determined by recording the response of MbNO on physiological solution using the Fe K-edge X-ray absorption time trace at 7127 keV, on the Fe K absorption edge.^3^ For the 200 µm-thick sample jet used for the XES measurements, a laser/X-ray correlation of σ = 150 ± 40 fs was determined, as can be seen from Supplementary Figure 16. Details about the fit can be found in Supplementary Notes 5 and 6.

The XES transients were recorded at a laser pulse energy of about 45 *μ*J, comparable to previous experiments on myoglobins,^3-5^ and corresponding to 2-3 photons per molecule. The spatial profile of the laser-beam at the sample position was found to be Gaussian with an effective spot diameter of 153x192 *μm^2^* (horizontal x vertical) using a beam profiler. The laser pointing fluctuations were characterized to be 2.77% horizontally and 3.65% vertically.

# Supplementary Note 3

**Steady-state X-ray emission spectroscopy (European XFEL):** Steady-state XES K_α_ and K_β_ reference spectra of [Fe(bpy)_3_]^2+^, deoxyMb and MbNO were recorded at the Eu-XFEL (see Supplementary Figures 3, 4 and 17), using the high-intensity X-ray pulses (pulse energy ~500 µJ) from the European XFEL. The ~100 fs pulses are delivered at a repetition rate of 30 pulses per train (at 10 Hz) and 1.1 MHz intra‐bunch train repetition rate.^6-8^ The intrinsically broad X-ray photon energy distribution (Δ*E*/*E* ≃ 10^−3^) was centered at 9.3 keV. The non-resonant steady-state X-ray emission spectra were recorded in the von Hamos geometry using seven Ge(220) crystals at a Bragg angle of θ_B_=75.45°for Fe K_α_, eight Si(531) crystals at θ_B_=73.09°for Fe K_β_ and a GreatEyes CCD(10 Hz) for detection. The X-ray (pink beam) was focused on the sample solution to a diameter of 40-50 *μ*m by beryllium refraction lenses. The sample-jet was placed in a small chamber filled with Nitrogen gas to prevent oxidation and a Helium bag was placed between the sample chamber, the crystals and the detector to reduce X-ray light attenuation in air.

# Supplementary Note 4

**Theoretical modelling (DFT):** Because K_α_ reference spectra of intermediate spin compounds are missing, Density functional theory (DFT) calculations were performed to simulate them. We benchmarked these calculations against the available MbNO and deoxyMb spectra (Supplementary Figure 4a). The calculations use the CAM-B3LYP exchange-correlation functional. Scalar relativistic effects were included using the second-order Douglas-Kroll-Hess Hamiltonian (DKH2), including picture change and finite-nuclear effects. The def2-TZVP basis set, reparametrized for use with DKH (DKH-def2-TZVP) was applied. Density fitting of the Coulomb and exchange integrals was employed using the resolution-of-the-identity chain-of-spheres approach (RIJCOSX), with the appropriate def2/JK auxiliary basis set. To approximate the effects of screening by the protein environment, a polarizable continuum model was applied using the conductor-like screening model (COSMO), using the same parameters as for toluene, in accordance with the low average dielectric constant within the interior of proteins.^9-11^ Spin-orbit coupling was included using the one-electron mean-field/effective potential approach. To ensure proper description of the core electrons, the accuracy of the radial integral grid in the vicinity of the Fe-atom was increased to Orca’s grid 7. All calculations were performed using Orca, version 3.0.3.^12^ The geometry employed in the calculations was based on the PDB entry 2FRJ for MbNO and dissociated, but not domed Mb calculations, for which the NO was removed, and PDB entry 2V1K for deoxyMb calculations. Supplementary Figure 1 overlays the structure of MbNO (red, PDB entry 2FRJ) and deoxyMb (green, PDB entry 2V1K) used in the present work. This highlights the main (as expected) structural differences that are probed in the ultrafast XES study.

Supplementary Figure 4b shows the DFT simulated static Kα XES for MbNO (doublet), deoxyMb (quintet) and deoxyMb (triplet) and compares them to experimental laser-on spectra in Supplementary Figure 4a. It is stressed that the DFT calculations do not reproduce absolute energies and the presented spectra are shifted for comparison to the experimental data. Furthermore, we do not reproduce the asymmetry on the red side of the spectral lines. This is due to the fact that the DFT functional used here does not fully account for multi-electron correlations, which are responsible for the sidebands, as shown in multiplet calculations.^13^ Therefore, while full line shapes do show deviations between theory and experiment, the relative changes reproduce the experimental trends, as seen in Supplementary Figure 14 where changes in peak intensity and shifts between the deoxyMb and MbNO experimental spectra are compared with the calculated ones.

We also simulated the Kα spectrum of the triplet state of deoxyMb, which is intermediate to the LS and HS cases (Supplementary Figure 4b). Laser-on Kα spectra at early time delays are shown in Figure 4. One notices a clear gradual weakening and a slight broadening of the bands within the first ps, after which the spectra no longer evolve. The experimental Kα XES transients at 0.26 and 1.36 ps time delay are shown in Supplementary Figure 9, while those at intermediate times and at later ones are shown in Supplementary Figure 10. The transient line shapes reflect the broadening and intensity changes of the asymmetric emission lines, which do not change beyond ~1 ps, by which time the system is in the HS state, as confirmed by the difference of experimental steady state spectra deoxyMb minus MbNO (Supplementary Figure 9). The difference of simulated triplet minus doublet and quintet minus doublet spectra, show a similar trend (Supplementary Figure 15) as the experimental transients, further supporting the conclusion of a transiently populated triplet state.

# Supplementary Note 5

**Determination of the instrument response function (IRF):** For the determination of the IRF, X-ray absorption spectroscopy (XAS) was used at the BL3 of SACLA. The intrinsically broad X-ray photon energy distribution was centered near the Fe K-edge by adjusting the conditions of the accelerator and undulators of SACLA, and a Si (111) X-ray monochromator, consisting of two channel-cut crystals with a (+,-,-,+) geometry^14^ was employed to scan the photon energy. The monochromatized probe X-ray was focused on the sample solution by beryllium compound refraction lenses to a focal beam diameter of 8 *μ*m.^14,15^ The XAS were measured in total fluorescence yield mode^16^ with a photodiode while scanning the monochromator. The sample-jet and the photodiode were placed in a box filled with Helium gas (>98%) to prevent sample oxidation and to reduce X-ray light attenuation in air. The intrinsic temporal jitter from the SASE operation is removed via post-processing of the measured data using the so-called timing tool, widely used at XFELs.^15^

The temporal instrument response function was determined from XAS pump-probe time scans of MbNO in physiological solution at 7127 eV*,* which is the main edge feature,^3^ shown in Figure Supplementary Figure 16. The rise of the signal at this energy was found to be IRF limited. It was fitted using the function described in Supplementary Note 6 (Supplementary Equation 1), yielding an IRF of 150 ± 40 fs that was then used to fit the XES kinetic traces.

# Supplementary Note 6

**Fit of the kinetic traces:** We are assuming a sequential kinetic scheme, for which the temporal evolution of the sample follows a sequence of events (rise or decays). These events can be described by specific first-order decay rates k_i_ and the corresponding lifetimes τ_i_ for which the kinetic response can be modeled by a sum of N model functions:^17^

$$\Delta A\left( t \right)=e^{-k_{i}t}\oplus\mathrm{IRF}\left( t \right)=$$

$$=\frac{1}{2}\sum_{i=1}^{N} a_{i}*e^{\frac{1}{\tau_{i}}*\left( \mu-t \right)+\frac{(\sigma*\frac{1}{\tau_{i}})^{2}}{2}}*$$

$*\left[ 1+erf\left( \frac{t-\left( \mu+\frac{1}{\tau_{i}}*\sigma^{2} \right)}{\sqrt{2}*\sigma} \right) \right]$ (1)

Where $a_{i}$ is the amplitude of the i^th^ decay, $\oplus$ indicates convolution, τ_i_ describes the decay time, which corresponds to $\frac{1}{k_{i}}$, µ the time origin of the IRF (time-zero) and σ its width.^17^ Combinations of N=1 to N=4 were fitted and the best fit was chosen according to least correlated lifetimes and amplitudes larger 0.01, as well as quality of the fits.

Each time-point of the XES time-traces is an average of three to six individual scans, one scan corresponds to accumulation over 10,000 XFEL shots. For the K_β_ XES, the line of the averaged, not smoothed, laser on (pumped) spectra (Figure 3) was fit with a Gaussian function for each time delay and its peak maximum (b in Supplementary Equation 2) was used to determine the shift in eV (Supplementary Figure 18):

$y=a*e^{\left[ -\left( \frac{x-b}{\sigma} \right)^{2} \right]}$ (2)

Where a is the amplitude, b is the centroid and σ is related to the peak width.

The obtained time trace was normalized to the maximum shift (~0.45 eV). For the K_α_ XES, the line was fitted with a Gaussian function (Supplementary Equation 2) to obtain σ, which was also normalized.

We are assuming a sequential kinetic scheme, for which the temporal evolution of the sample follows a sequence of events (rise or decays). These events can be described by specific first-order decay rates k_i_ and the corresponding lifetimes τ_i_ for which the kinetic response can be modeled by a sum of N model functions.^17^

# Supplementary Note 7

**Estimation of the photodissociation quantum yield:** To obtain meaningful difference spectra for K_β_ XES, we first estimate the photodissociation quantum yield and use it in the section below to calculate the difference spectra for comparison with our results. Considering the absorbance of MbNO in physiological solution at 533 nm (Supplementary Figure 2), we obtain a ratio of photons per molecule of 2-3 and therefore assume that each MbNO absorbs at least one photon. From the K_β_ reference spectra of different spin states from Zhang et al,^18^ one can obtain a shift of 1.6 eV for $K_{\beta_{1,3}}$ for a spin change from doublet to quintet state. For the case of MbNO this would mean that in the case of a photodissociation quantum yield of 100% we would expect the same energy shift. Taking the laser-on spectrum of MbNO at 1.36 ps delay, we observe a shift of ~0.45 eV for the K_β1,3_ line. Trying different ratios of doublet:quintet for the reference spectra from Zhang et al,^18^ we obtain a shift of 0.45 eV from the doublet to doublet/quintet spectra with a ratio of 45:55 (doublet/quintet), which matches best in terms of peak shift and broadening and corresponds to a photodissociation quantum yield of 55%. This is in good agreement with the 50 ± 5% reported in the literature^19^ and is therefore used in the next section to calculate the XES K_α_ and K_β_ difference spectra to be compared to our transient spectra for MbNO.

# Supplementary Note 8

**Derivation of the K_α_ and K_β_ difference spectra for comparison:** The triplet and quintet difference spectra in Supplementary Figure 7 are derived using the reference spectra from ref. ^18^ shown in Supplementary Figure 3. For the triplet state the XES K_β_ spectrum of Iron(ii)-phthalocyanine was used (I_triplet_). The [Fe(phenanthroline)_2_(NCS)_2_] XES K_β_ spectrum represents the quintet state spectrum (I_quintet_) and for the doublet ground state, the XES K_β_ spectrum of [Fe(2,2′-bipyridine)_3_]^3+^ was used. First the excited state spectra are calculated using a photodissociation quantum yield of 55%:

$\mathrm{ES}_{\mathrm{quintet}}=0.45*I_{\mathrm{doublet}}+0.55*I_{\mathrm{quintet}}$ (3)

ES_quintet_ corresponds to the expected excited state spectrum for MbNO (deoxyMb) (quintet excited state), I_doublet_ are the intensity values of the spectrum of ([Fe(bpy)_3_]^3+^) representing 100% doublet state and I_quintet_ are the intensity values of the spectrum of ([Fe(Phen)_2_(NCS)_2_]) representing 100% quintet state. Afterwards the difference spectra are calculated:

$\mathrm{quintet}_{\mathrm{difference}}=\mathrm{ES}_{\mathrm{quintet}}-I_{\mathrm{doublet}}$ (4)

to obtain the quintet difference spectrum ($\mathrm{quintet}_{\mathrm{difference}}$). For the triplet state pump-probe spectrum the same was done using the triplet state XES K_β_ spectrum of (iron(ii) phthalocyanine) from ref. ^18^. The same procedure was used to obtain the triplet and quintet K_α_ transient spectra in Supplementary Figure 15 using DFT simulated spectra (see Supplementary Note 4) since experimental reference spectra are lacking for the K_α_ emission.

# Supplementary Note 9

**Error analysis:** The uncertainties on all time constants represent the 1-sigma uncertainty. The error bars in the time traces represent the 1-sigma uncertainty of the Gaussian fit.

All XES K_β_ data and the XES K_α_ transients at 0.26 ps presented in this work are smoothed using a 5-point moving average filter to make trends more visible. The first few elements of yy follow:

yy(1) = y(1)

yy(2) = (y(1) + y(2) + y(3))/3

yy(3) = (y(1) + y(2) + y(3) + y(4) + y(5))/5

yy(4) = (y(2) + y(3) + y(4) + y(5) + y(6))/5

...

Supplementary Figures 17 and 18 show the XES K_β_ and K_α_ spectra, respectively, with their error bars, calculated from the standard deviation of the scans acquired. This clearly shows that the observed trends are larger than the noise of the experiment.

# Supplementary Note 10

**Kinetic model:** To estimate the decay rate from the excited intermediate (int.) state to the quintet state using the K_β_ transient spectra in the time range of 0.26 to 3.16 ps (Supplementary Figure 6), we use a simple 3-level kinetic model (Supplementary Figure 11), with decay rates of *Γ*_LUMO→int._ and *Γ*_triplet→int._, for the Q-state (LUMO) of the porphyrin and triplet levels, respectively. The master equation of the model is

$\frac{d}{dt}\left( \begin{matrix} \rho_{Q-state(t)} \\ \rho_{Int.(t)} \\ \rho_{Quintet(t)} \end{matrix} \right)=\Gamma*\left( \begin{matrix} \rho_{Q-state(t)} \\ \rho_{Int.(t)} \\ \rho_{Quintet(t)} \end{matrix} \right)$ (5)

Where Γ is defined by

$\Gamma=\left( \begin{matrix} {-\Gamma}_{Q-state\to Int.} & 0 & 0 \\ \Gamma_{Q-state\to Int.} & {-\Gamma}_{Int.\to Quintet} & 0 \\ 0 & \Gamma_{Int.\to Quintet} & 0 \end{matrix} \right)$ (6)

From fluorescence up-conversion measurements $\left( \Gamma_{Q-state\to Int.} \right)^{-1}\leq100 fs$. ^20^

The boundary conditions are: 100% Q-state population, 0% population of the Intermediate (Int.) and quintet state population at t=0. Solving the system of differential equations provides the time evolution of $\rho_{Int.}\left( t \right)$.

$\rho_{Int.}(t)=\frac{\Gamma_{L}}{\Gamma_{L}-\Gamma_{I}}*\left( e^{-t*\Gamma_{I}}-e^{-t*\Gamma_{L}} \right)$ (7)

$\rho_{\mathrm{Quintet}}(t)=\frac{\Gamma_{L}}{\Gamma_{L}-\Gamma_{I}}*\left( 1-e^{-t*\Gamma_{I}} \right)-\frac{\Gamma_{I}}{\Gamma_{L}-\Gamma_{I}}*\left( 1-e^{-t*\Gamma_{L}} \right)$ (8)

with $\Gamma_{L}=\Gamma_{Q-state\to Intermediate}$ and $\Gamma_{I}=\Gamma_{Intermediate\to Quintet}$.

The data points are the mean of seven points (7053.0 to 7054.0 eV) in Supplementary Figure 6, which are offset and an artificial point with 0 intensity before time zero was added to allow a reasonable fit. These means are plotted in Supplementary Figure 12 and fitted assuming that the relaxation after t_0_ is proportional to the population of the intermediate state. The derived intermediate to quintet relaxation time is τ_intermediate→quintet_ = 500± 250 fs. Supplementary Figure 10 shows the fit together with the 90% confidence bands. Supplementary Figure 13 shows the evolution of Q-state, intermediate and quintet populations derived from the kinetic model. It can be seen that the quintet state rises in approximately 700 fs.


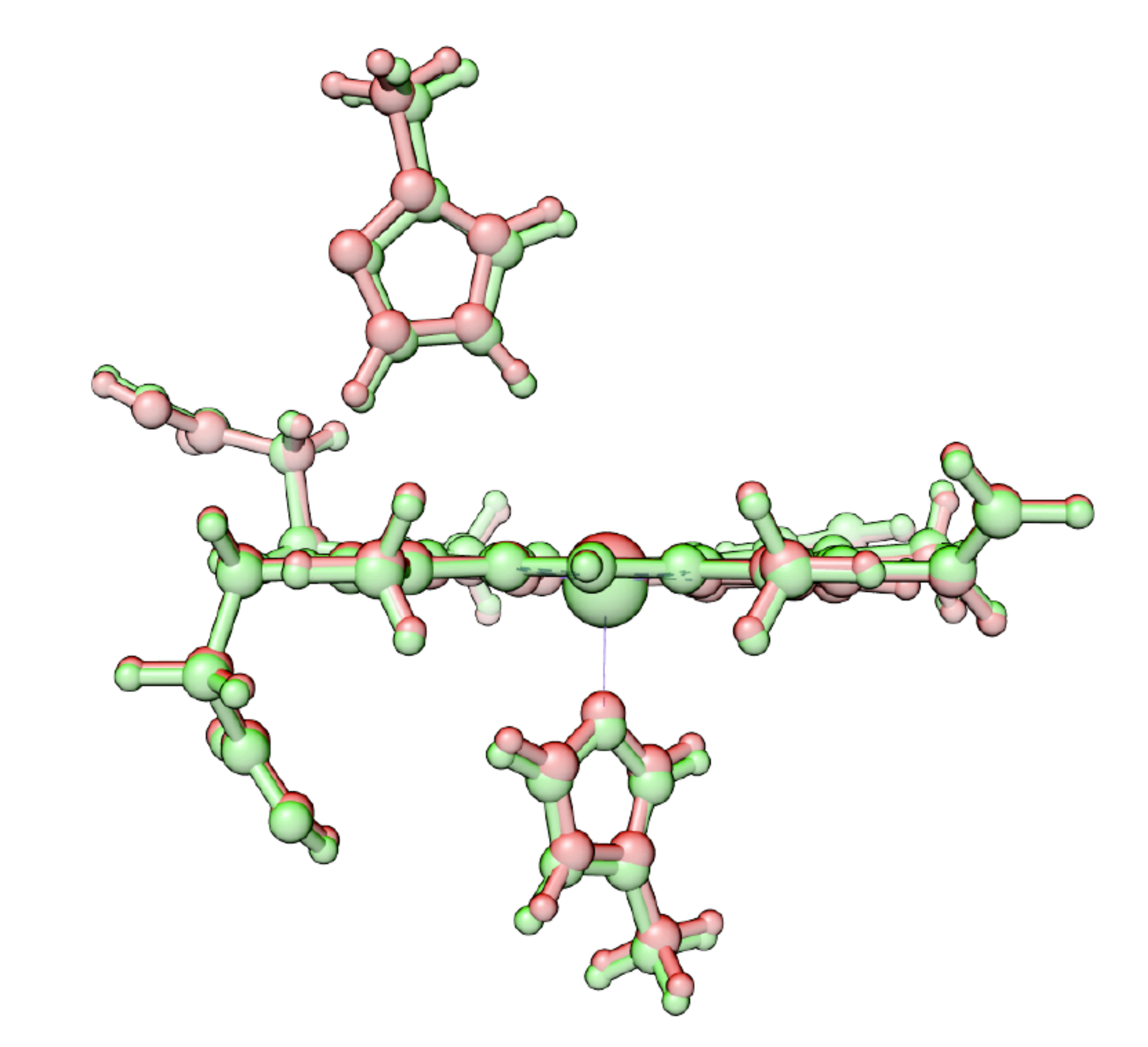


Supplementary Figure 1: Overlaid structure of MbNO without NO (red, PDB entry 2FRJ) and of deoxyMb (green, PDB entry 2V1K).

Supplementary Figure 2: Absorption spectra of 4mM solutions of MbNO and deoxyMb (in a 100 µm quartz flow cell) in the region of the Q bands.


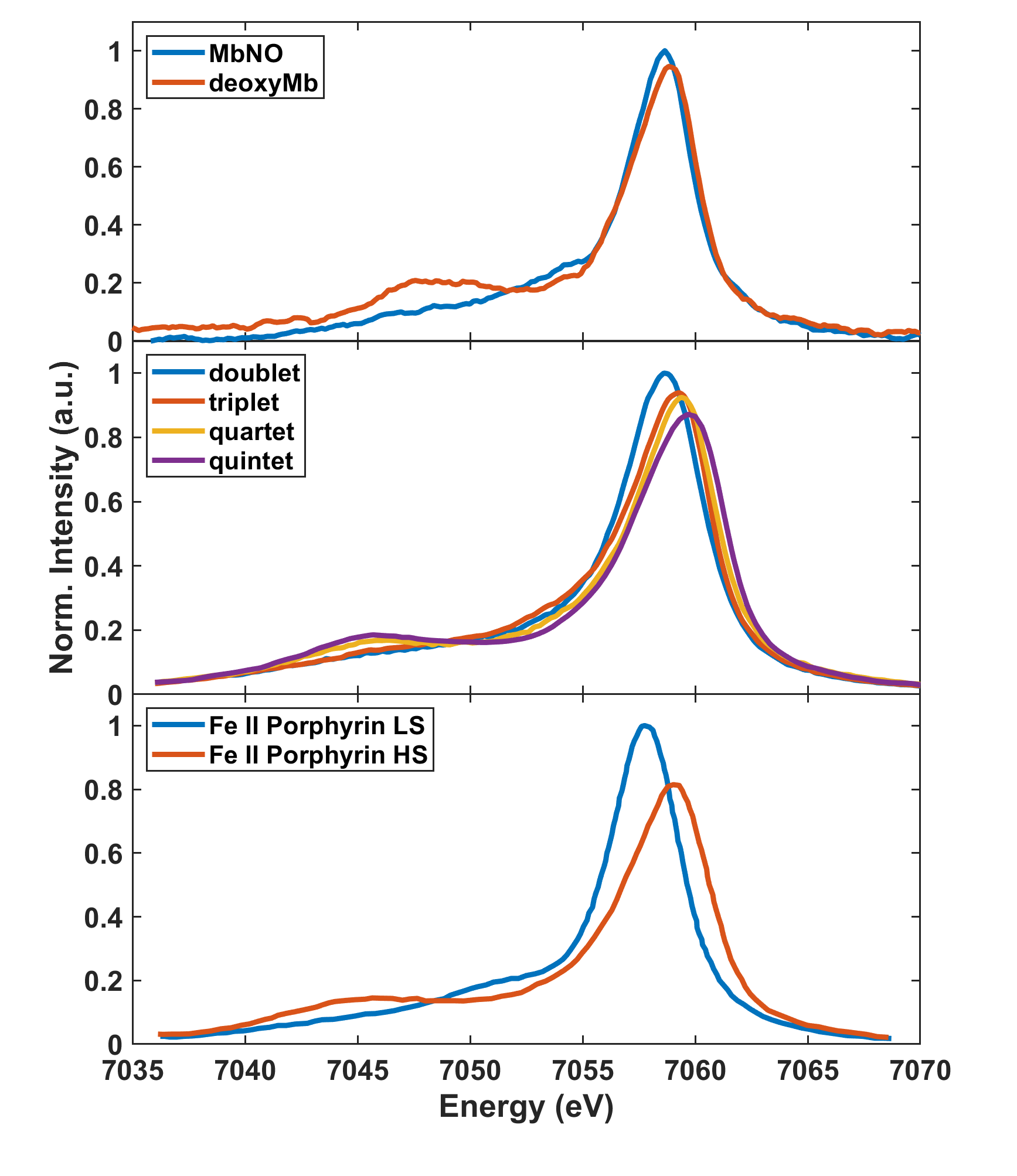


**c)**

**b)**

**a)**

Supplementary Figure 3: XES K_β_ spectra and references a) Steady-state XES K_β_ spectra of deoxyMb (S=2) and MbNO (S=1/2) measured at Eu-XFEL and SACLA, relative to the peak maximum of MbNO normalized to 1. b) Reference XES K_β_ spectra of ground state [Fe(2,2′-bipyridine)_3_]^3+^ (S=1/2, blue), triplet state (iron(ii) phthalocyanine, green), quartet state (iron(iii) phthalocyanine chloride, yellow) and [Fe(phenanthroline)_2_(NCS)_2_] (S=2, red) reproduced from ref. ^18^. c) Normalized XES K_β_ reference spectra at 260 K of model porphyrin complexes Fe^II^(TPP)(Py)_2_ (LS) and Fe^II^ (TPP)(THF)_2_ (HS) (TPP = tetraphenylporphyrin, Py = pyridine, THF = tetrahydrofuran), reproduced from ref. ^21^.


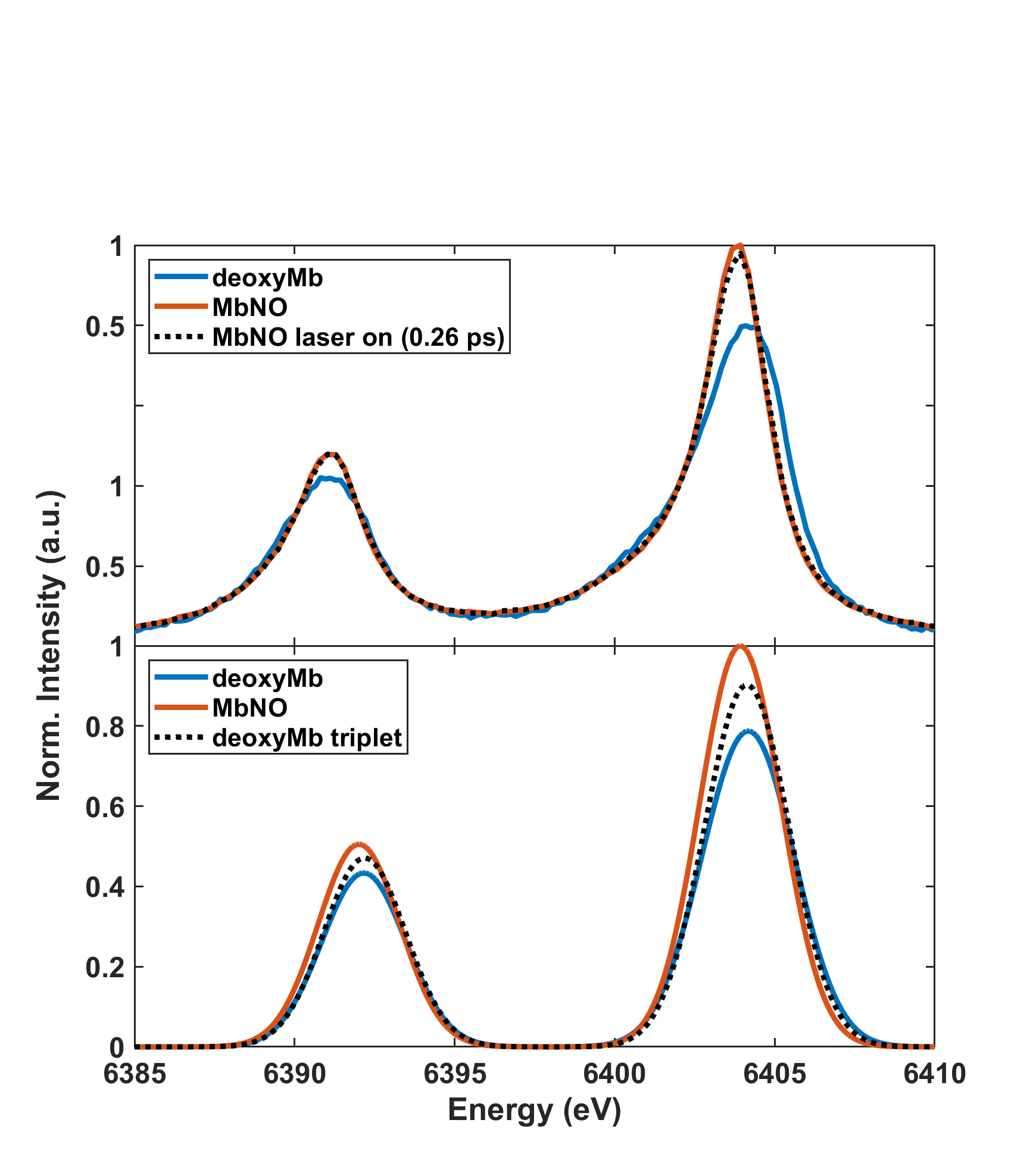


**b)**

**a)**

Supplementary Figure 4: a) Experimental Fe Kα spectra of ground state deoxyMb (S=2) and MbNO (S=1/2) and of the laser-on MbNO spectrum at 0.26 ps after excitation, all normalized relative to the peak maximum of the ground state MbNO spectrum. The Kα1 line is near 6404 eV and the Kα2 line is near 6391 eV. b) DFT simulated XES Kα spectra of deoxyMb, MbNO and deoxyMb triplet state, all shifted by +86.3 eV and normalized relative to the peak maximum of the MbNO spectrum. In (a), the 0.26 ps contains both the XES of the ground state MbNO and of the triplet deoxyMb.


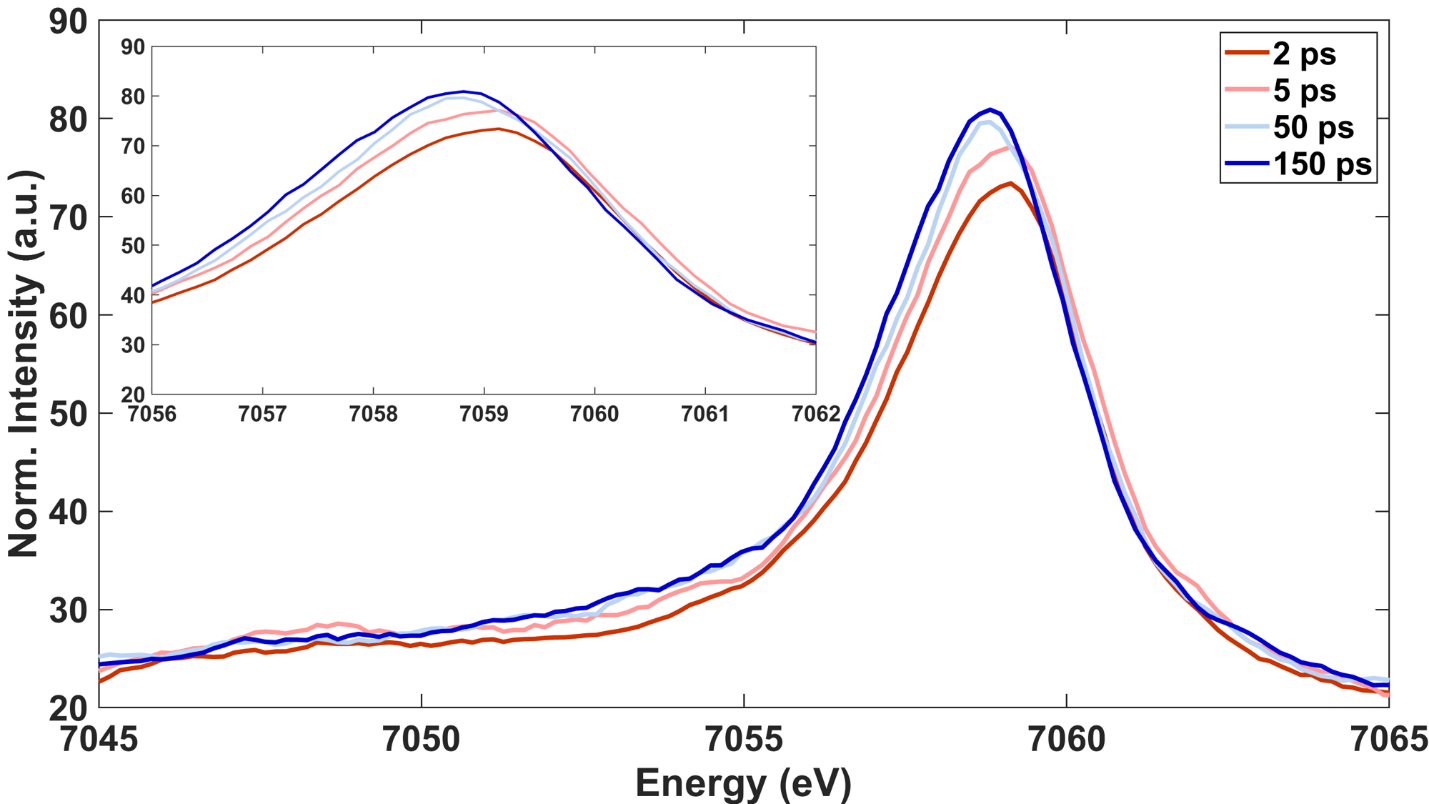


Supplementary Figure 5: Laser-on (pumped) K_β_ XES spectra of MbNO at different time delays between 2 and 150 ps (from red to blue) showing a red shift of the K_β1,3_ line and an intensity increase. The inset zooms the region of the maximum of $K_{\beta_{1,3}}XES$line at different time delays between 2 and 150 ps (from red to blue) showing peak shifts smaller than the energy resolution (~0.5 eV) of our experiment.


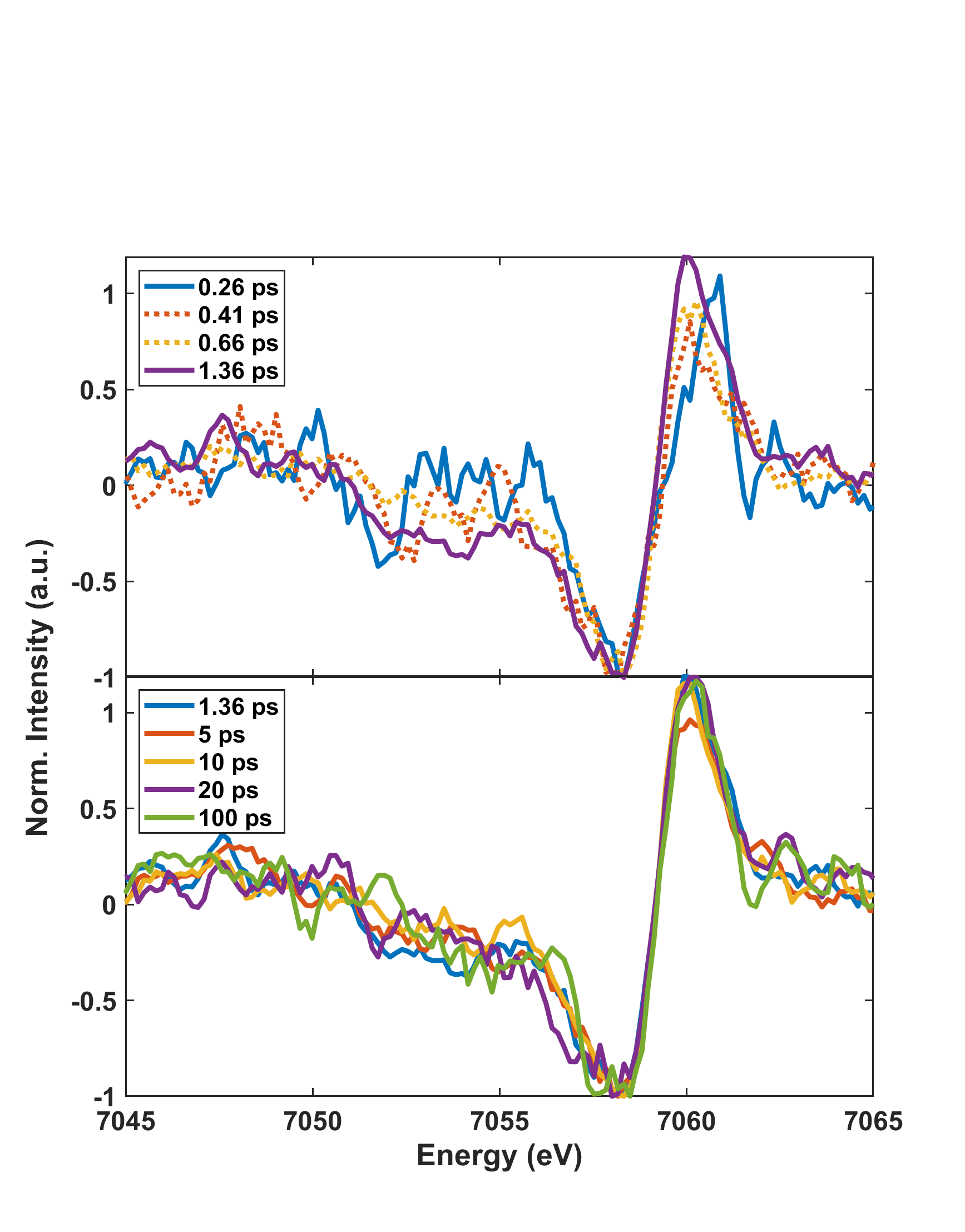


**b)**

**a)**

Supplementary Figure 6: a) Transient K_β_ XES spectra of photoexcited MbNO between 0.26 and 1.36 ps (normalized to the maximum of the negative signal). b) same for transients between 1.36 and 100 ps.


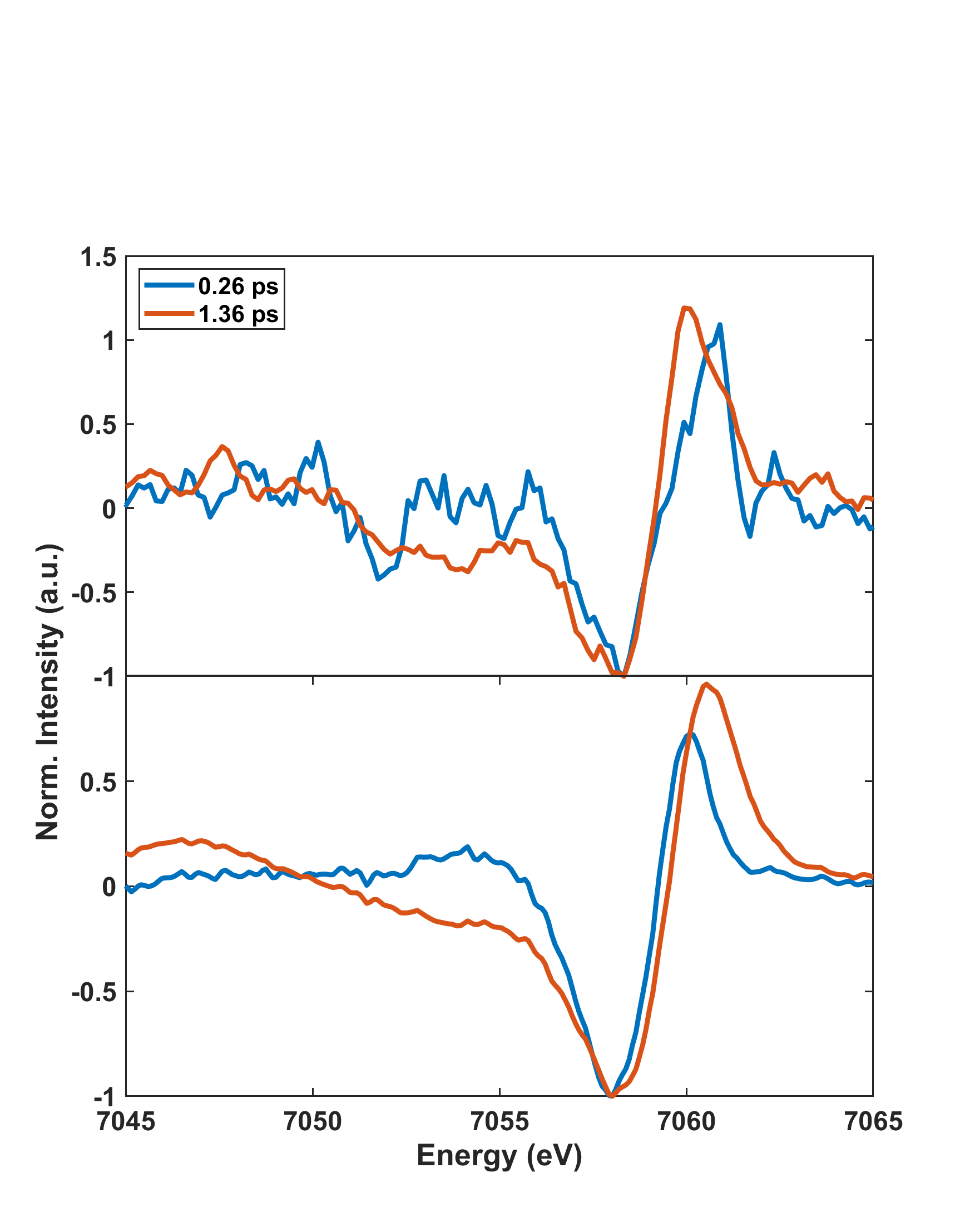


**b)**

**a)**

Supplementary Figure 7: a) Transient XES K_β_ spectra of MbNO in a physiological solution at pump-probe time delays of 0.26 and 1.36 ps. b) Difference of steady-state XES K_β_ spectra from ref. ^18^: triplet minus doublet (blue), quintet minus doublet (red), using the reference spectra of Fe(II)phthalocyanine (triplet state), [Fe(2,2′-bipyridine)_3_]^3+^ (doublet state) and [Fe(phenanthroline)_2_(NCS)_2_] (quintet state), and taking into account NO dissociation quantum yield (Details can be found in Supplementary Note 8).


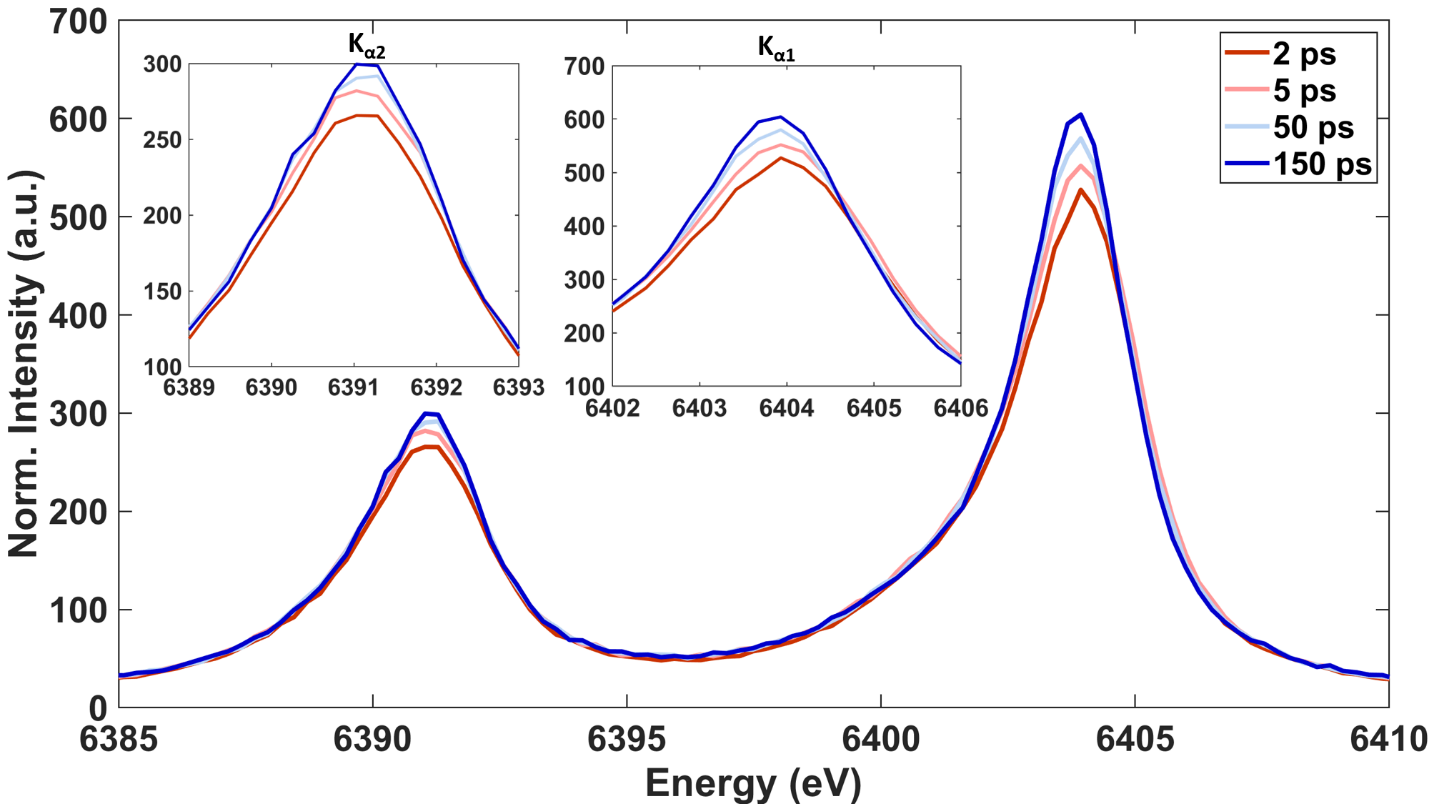


Supplementary Figure 8: Laser-on (pumped) $K_{\alpha}$XES spectra of MbNO at different time delays between 2 and 150 ps (from red to blue) showing an intensity increase for K_α2_ and an intensity decrease and change in peak width for K_α1_. Also, the visibility of changes in peak width (σ) smaller than the energy resolution (~0.6 eV) can be well observed. The insets zoom into the peaks of the K_α2_ and K_α1_ laser-on (pumped) XES spectra of MbNO at different time delays between 2 and 150 ps (from red to blue) showing an intensity increase for K_α2_ and an intensity increase and change in peak width for K_α1_. The changes in peak width (σ) smaller than the energy resolution (~0.6 eV) can be well observed.


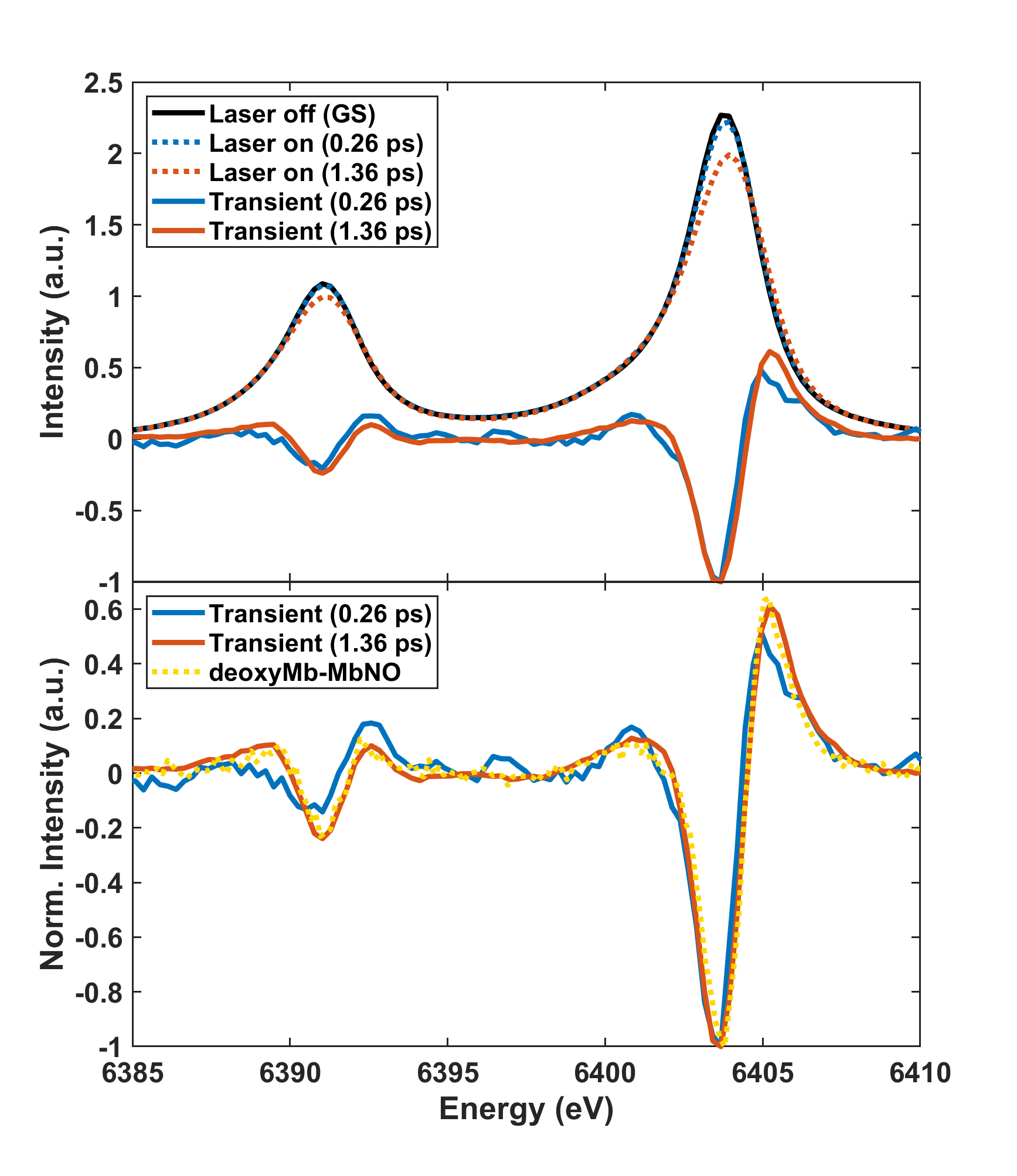


**b)**

**a)**

Supplementary Figure 9: (a) Ground-state K_α_ spectrum of MbNO (black) and Laser-on (pumped) spectra at 0.26 ps (blue), representing mainly triplet state population and at 1.36 ps (red), representing mainly quintet state population. (b) Transient XES K_α_ spectra at 0.26 and 1.36 ps showing a blue shift of the positive K_α1_ transient feature (6405 eV) from 0.26 ps to 1.36 ps, and the presence of a positive dip at 6393 eV at 0.26 ps, which vanishes for later times (1.36 ps). In yellow (dotted) the static difference signal of deoxyMb-MbNO is plotted, which resembles the spectrum at 1.36 ps. This indicates structural and electronic similarity of the excited state after ~1 ps and the deoxyMb.


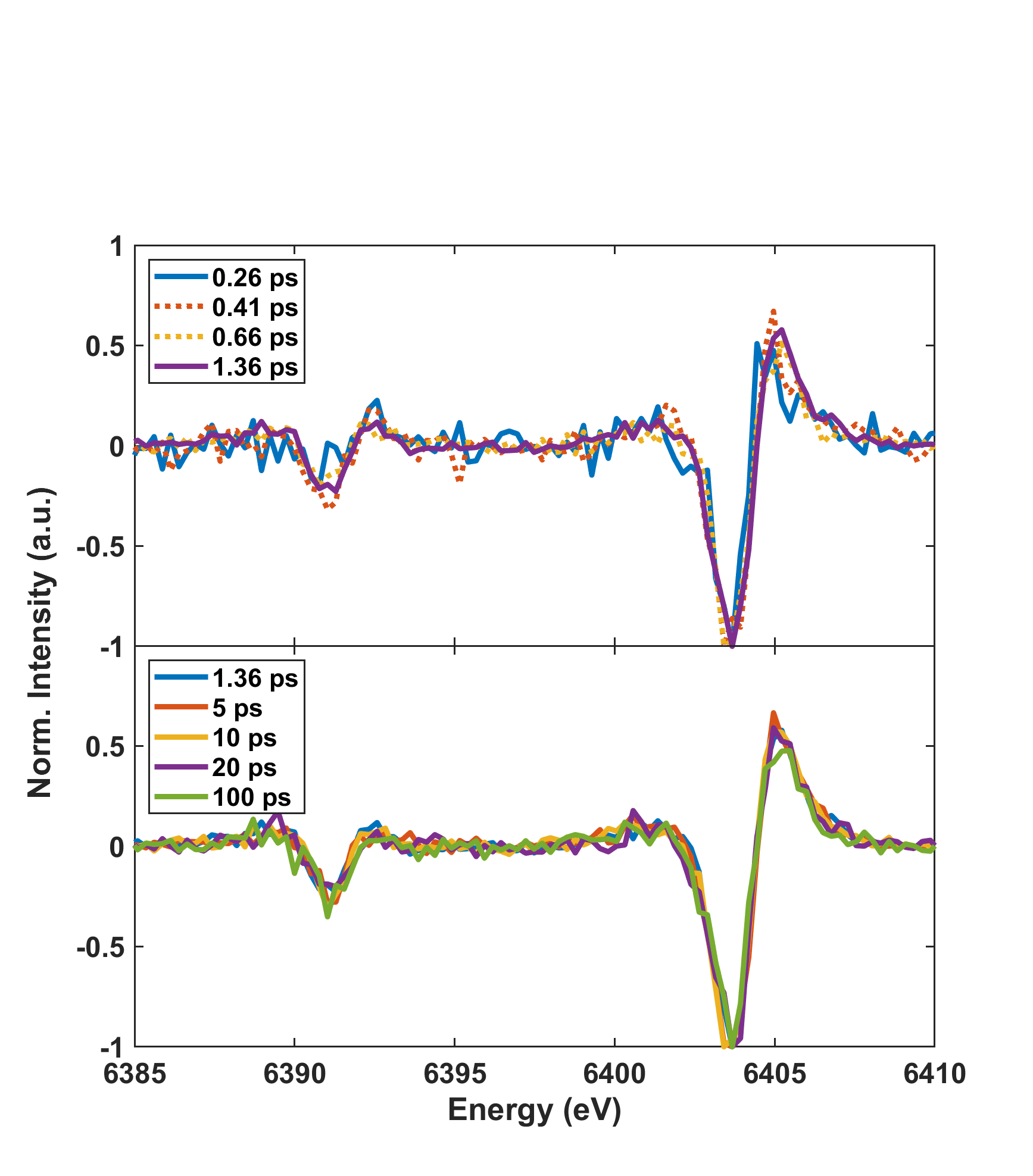


**b)**

**a)**

Supplementary Figure 10: a) Transient K_α_ XES spectra of MbNO between 0.26 and 1.36 ps (normalized to the maximum of the negative signal). b) same for transients between 1.36 and 100 ps.


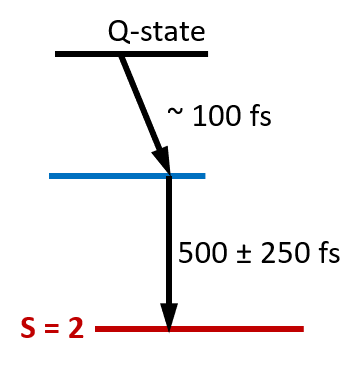


Supplementary Figure 11: Energy level diagram showing the LS (S=1/2) Q-state (LUMO) of the porphyrin, the intermediate (blue) and the S=2 states with arrows indicating the relaxation pathway and their corresponding rates. The ~100 fs decay of the Q-state is taken from fluorescence up-conversion measurements,^20^ the 500 ± 250 fs is derived from the kinetic model (see Supplementary Note 10).


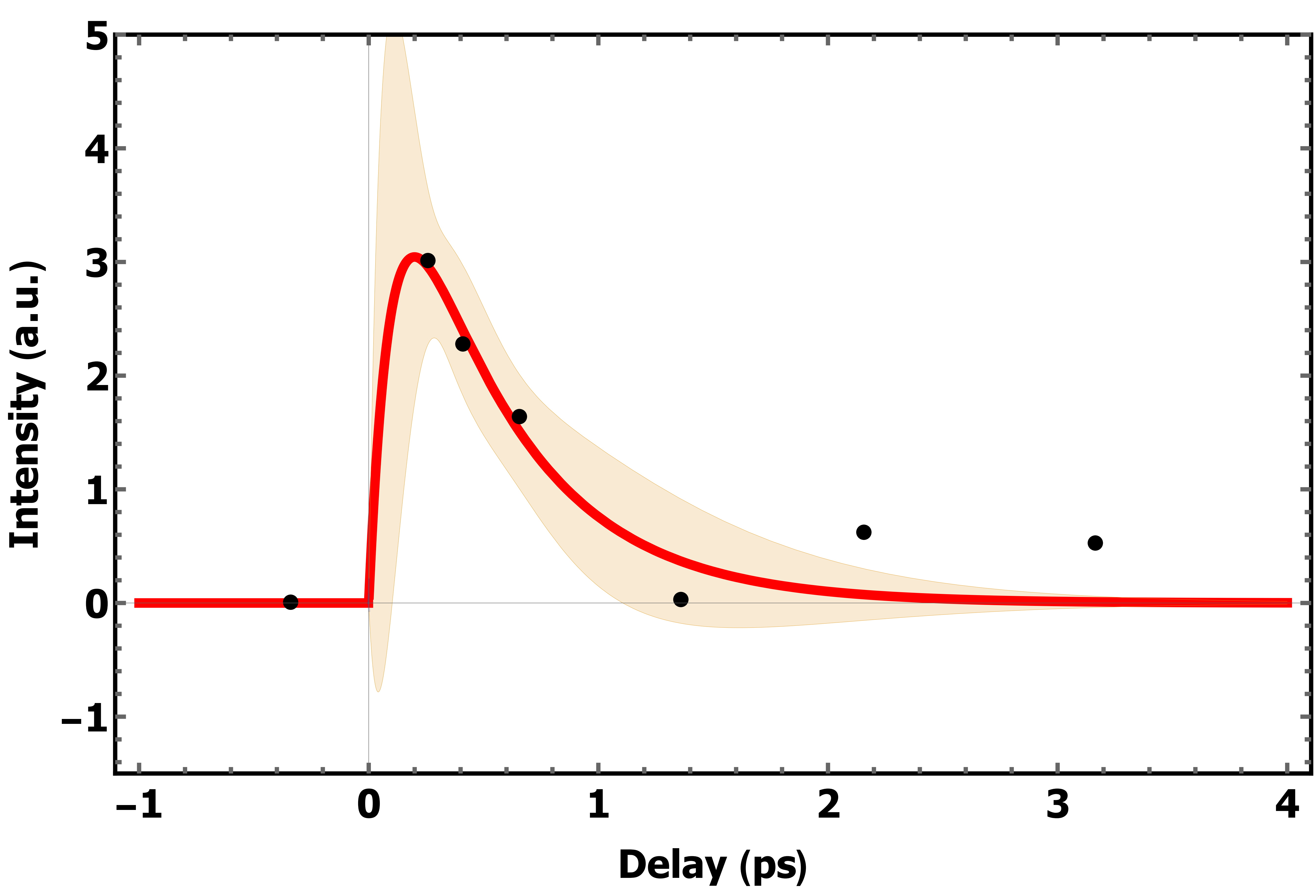


Supplementary Figure 12: Plotted are the mean of seven points (7053.0 to 7054.0 eV) of the K_β_ XES pump-probe spectra (Supplementary Figure 6) in the time range of 0.26 to 3.16 ps (black dots). and the fit (red) together with the 90% confidence bands (orange shade). The data points are offset and an artificial point with 0 intensity before time zero was added to allow a reasonable fit. See Supplementary Note 10 for details about the kinetic model.


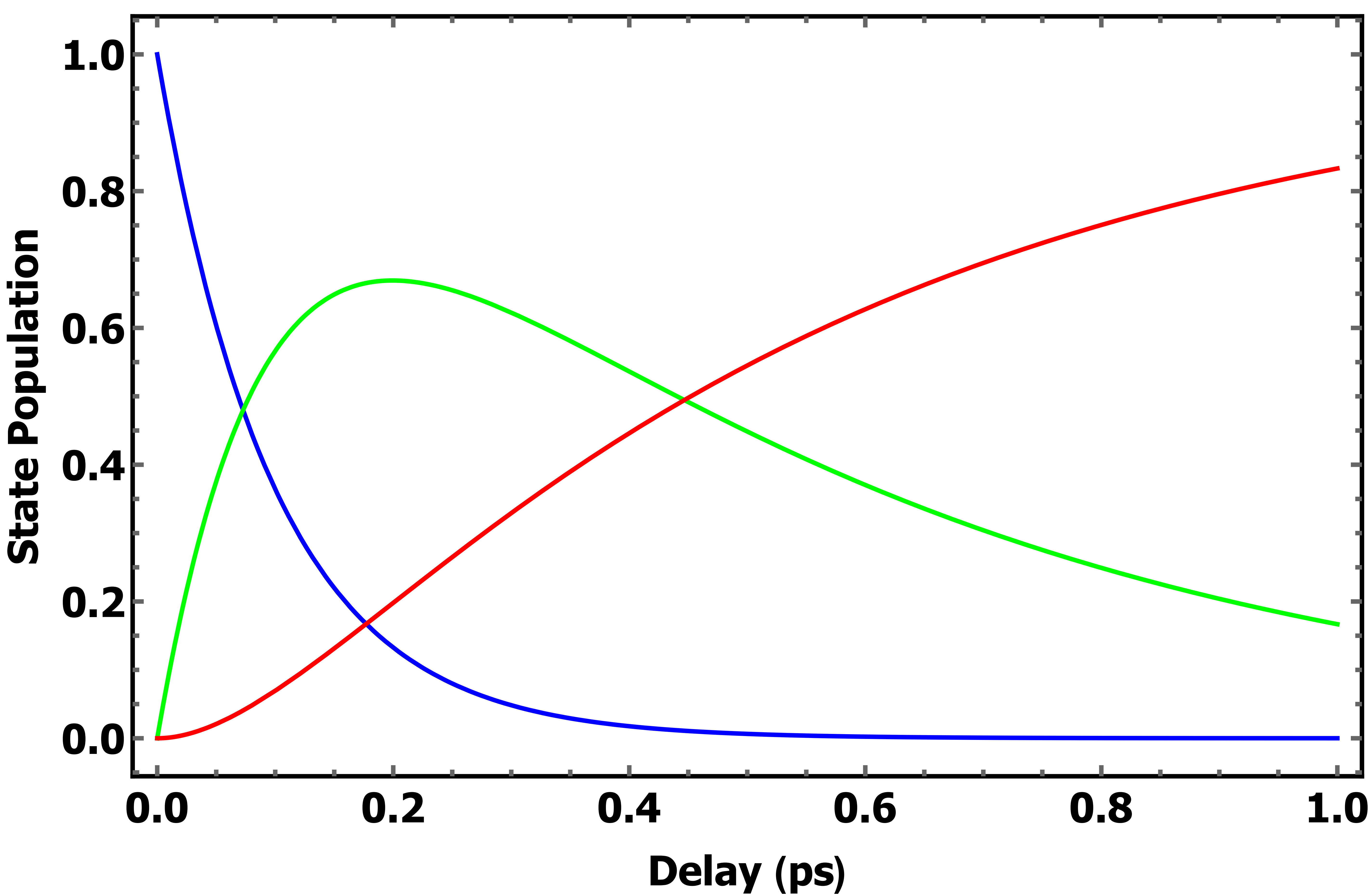


Supplementary Figure 13: Displays the population dynamics obtained from the kinetic model (see Supplementary Note 11) after t_0_ of ρ_Q-state_[t] (blue), ρ_Int._[t] (green) and ρ_Quintet_[t] (red).


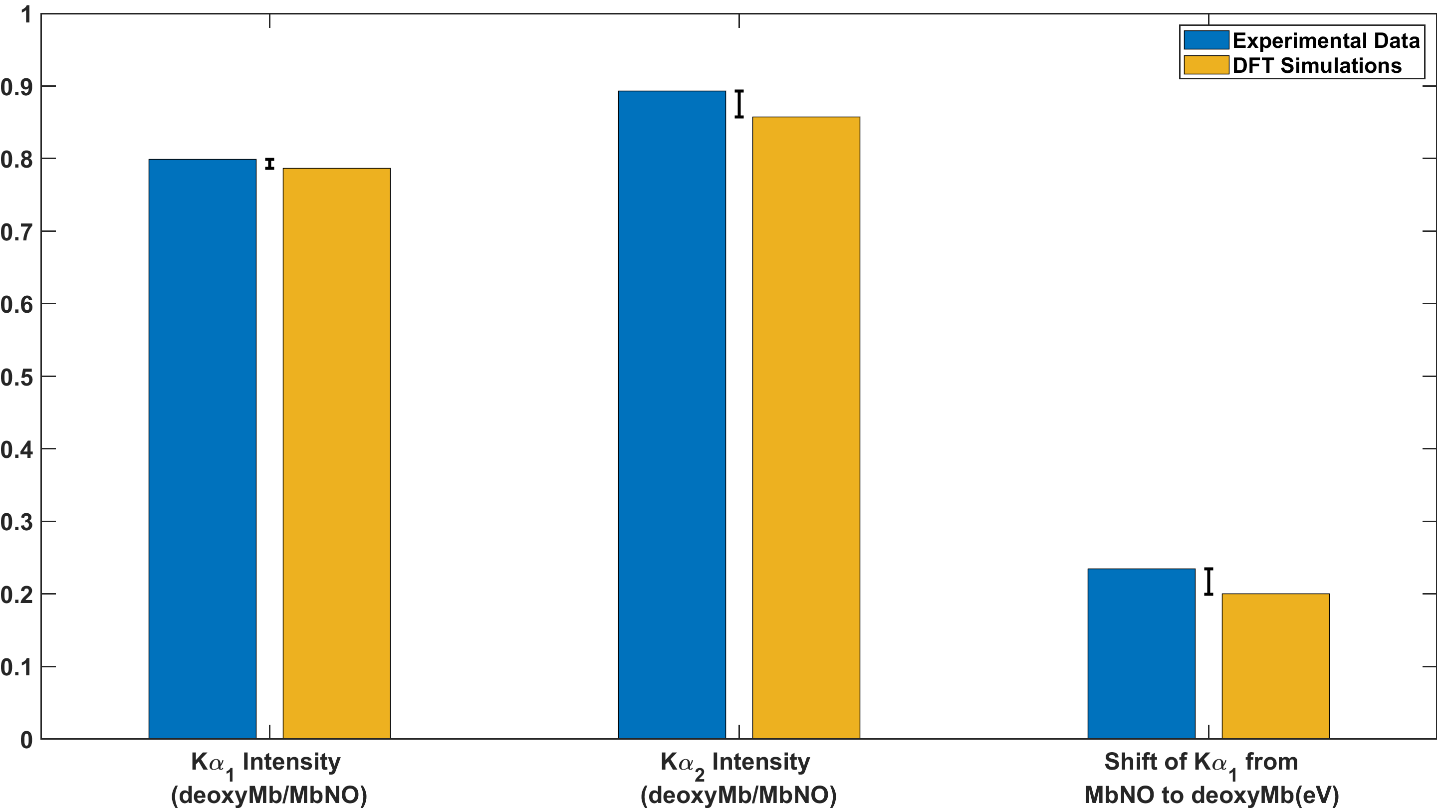


Supplementary Figure 14: Comparison of relative ratios between deoxyMb and MbNO of the K_α1_ intensity (left), the K_α2_ intensity (middle) and the shift of K_α1_ from deoxyMb to MbNO for experimental (blue) and DFT (orange) XES Kα spectra and the standard-deviation (STD) between the experimental results and DFT simulations are shown in between. This serves as benchmark of the DFT simulations, showing that relative changes in the spectra reproduce well the experimental trends.


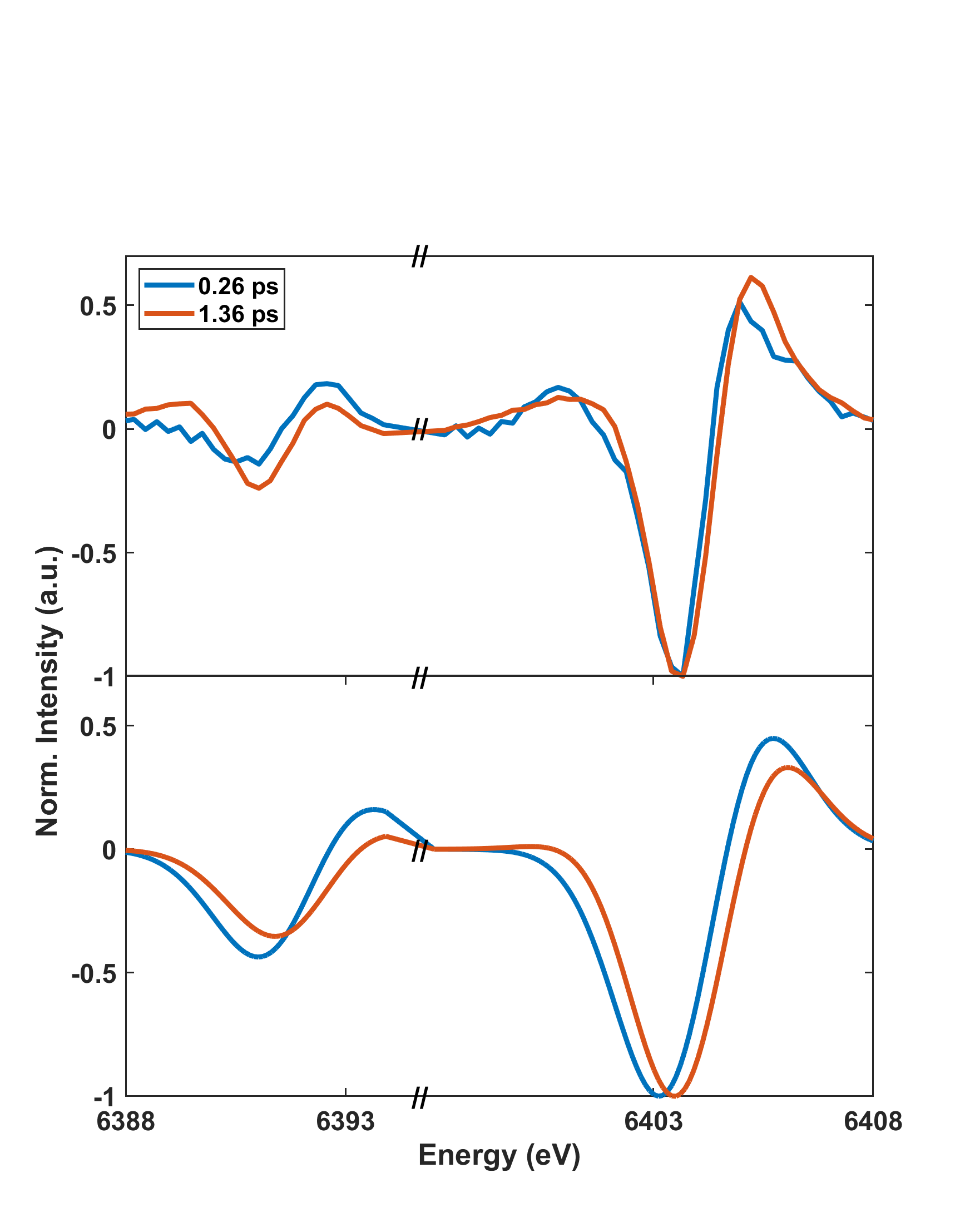


**b)**

**a)**

Supplementary Figure 15: (a) Transient XES K_α_ spectra at 0.26 and 1.36 ps showing a blue shift of the positive K_α1_ transient feature (6405 eV) from 0.26 ps to 1.36 ps, and the presence of a positive dip at 6393 eV at 0.26 ps, which vanishes for later times (1.36 ps). (b) XES K_α_ difference spectra derived from DFT simulated XES K_α_ spectra: triplet minus doublet (blue), quintet minus doublet (red). Details can be found in Supplementary Note 8.


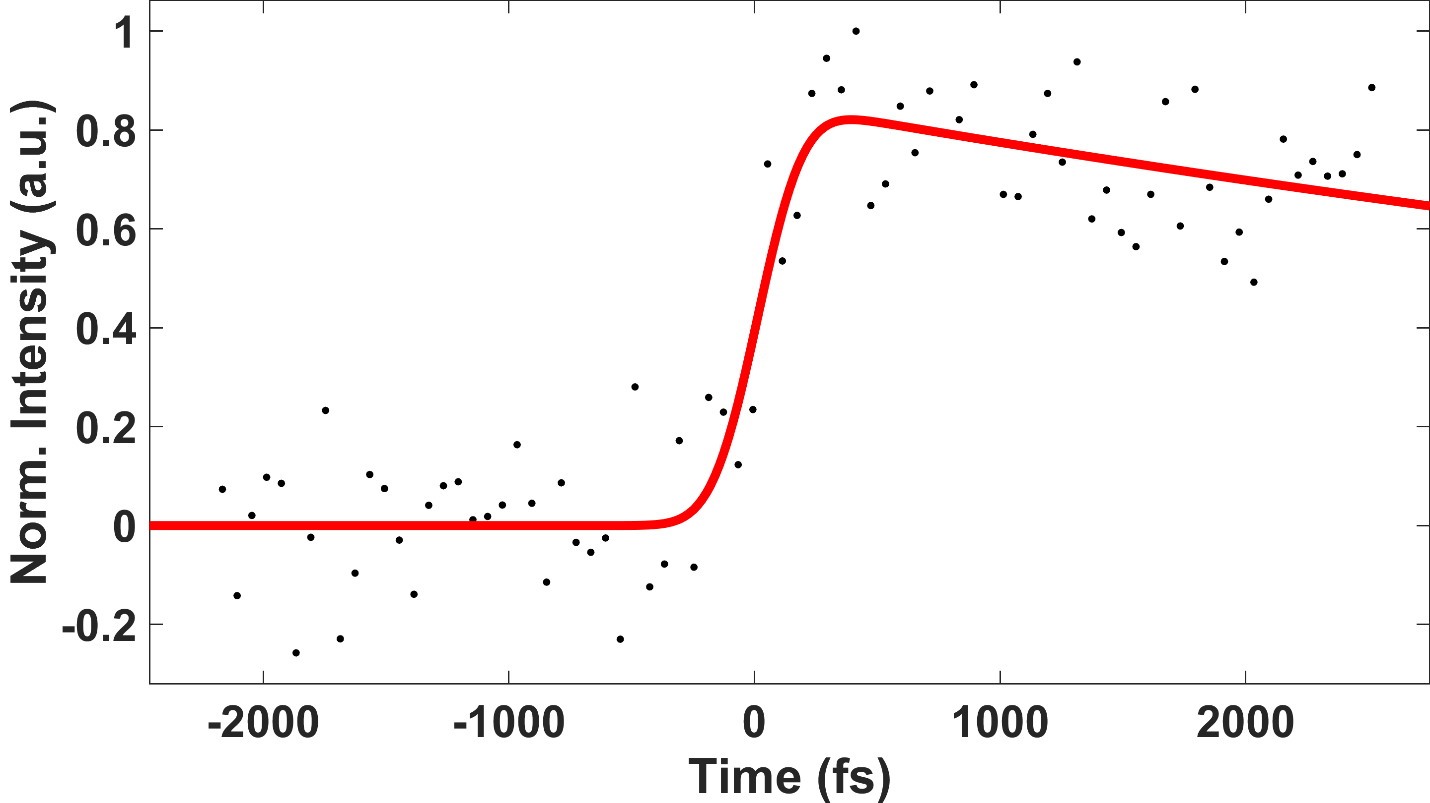


Supplementary Figure 16: Rise time of the X-ray absorption transient signal of MbNO at 7127 eV (dots). The red trace is a fit to obtain the IRF of the experiment (see Supplementary Note 6), which is σ = 150 fs.


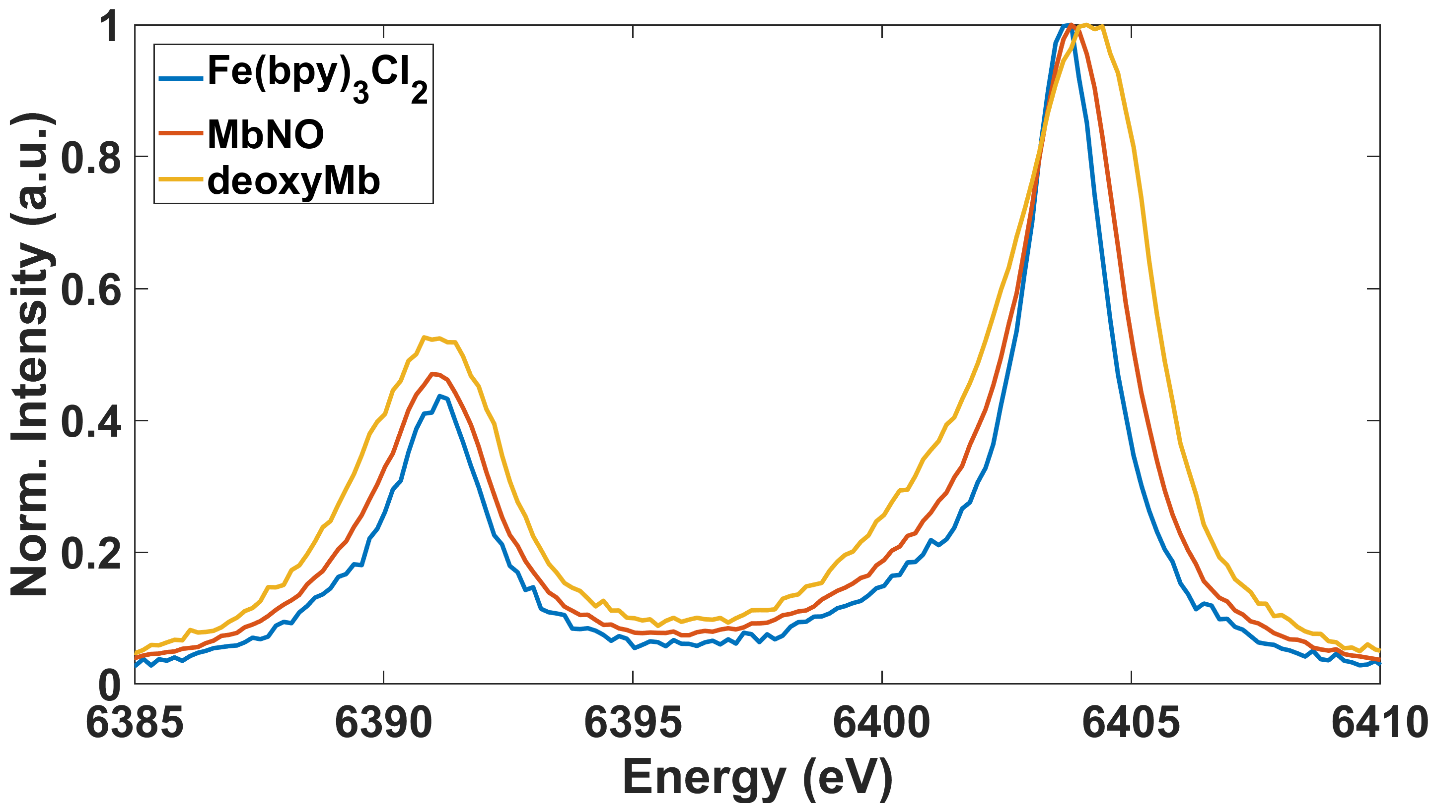


Supplementary Figure 17: XES K_α_ spectra normalized to the maximum peak intensity for singlet (Fe(bpy)_3_Cl_2_), doublet (MbNO) and quintet (deoxyMb) compounds.


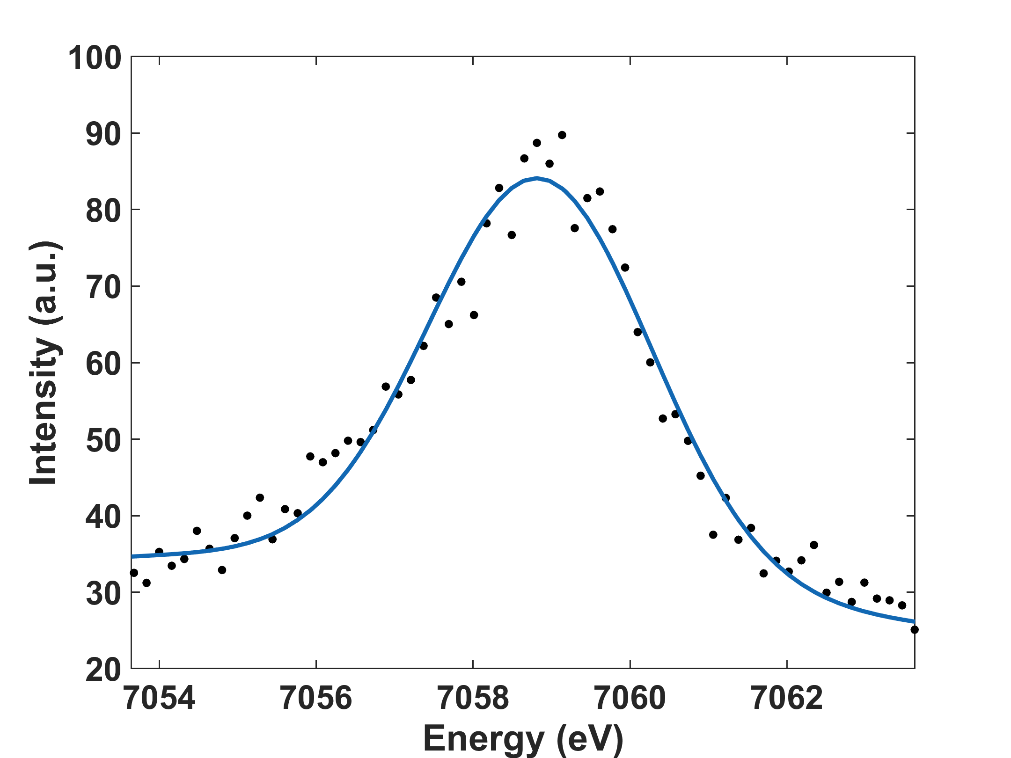


Supplementary Figure 18: XES K_β_ laser on spectra at 10 ps delay measured at SACLA (black dots). In blue the Gaussian fitting used to obtain the peak maximum. The error bars in the time traces represent the 1-sigma uncertainty of the Gaussian fit.


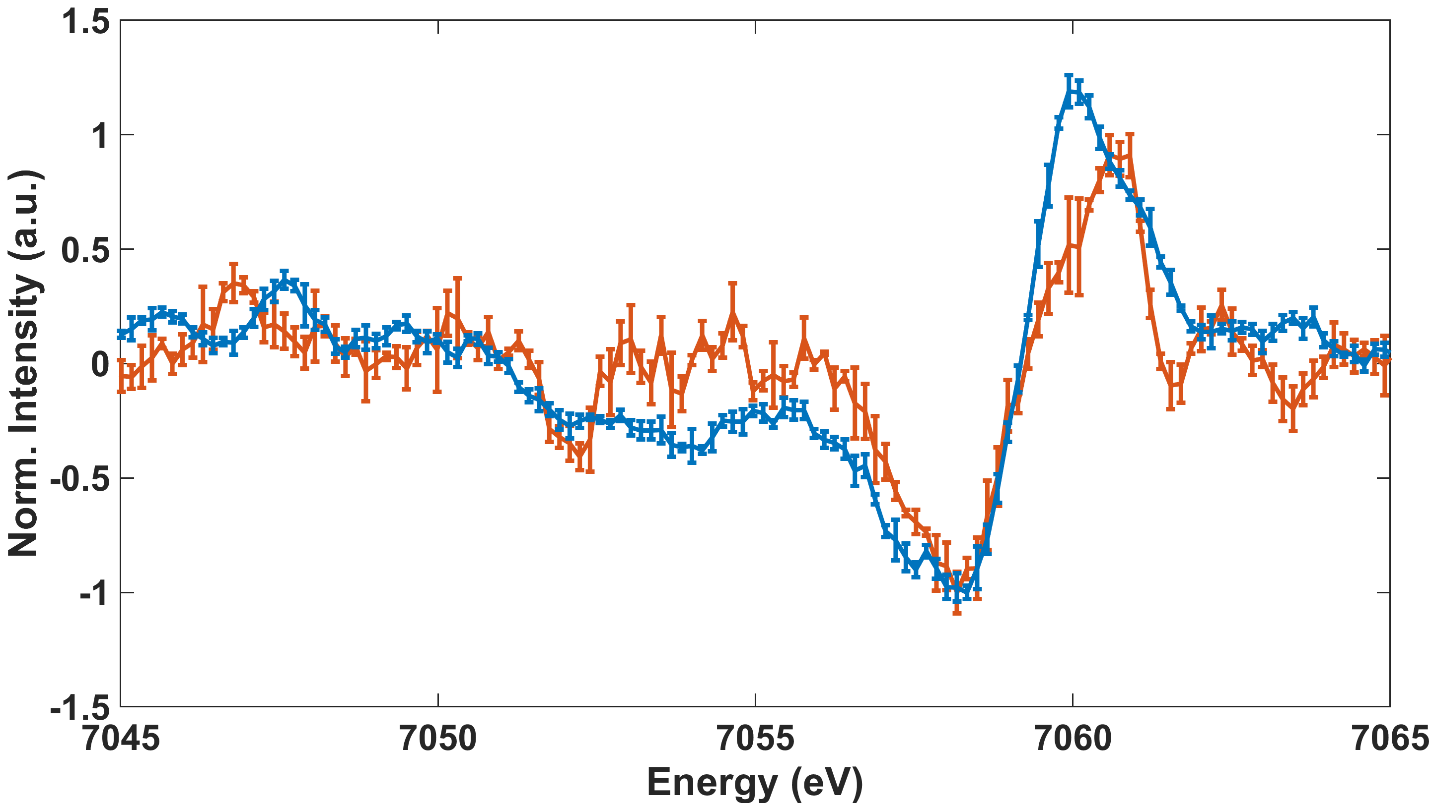


Supplementary Figure 19: Transient K_β_ XES spectra of MbNO at 0.26 (red) and 1.36 ps (blue). The 0.26 ps spectrum represents predominantly triplet state species and the 1.36 ps spectrum the quintet state. Both are normalized to the maximum of the negative signal. The error bars are the standard deviation between the runs.


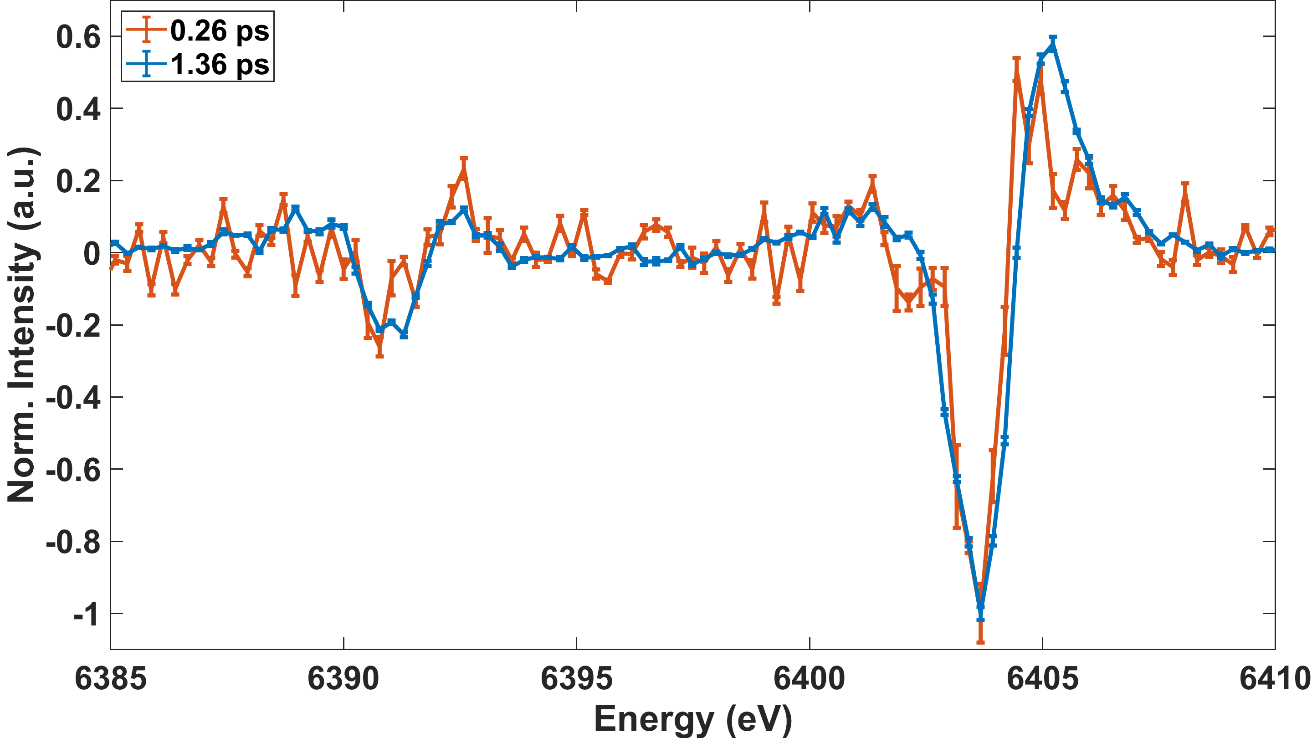


Supplementary Figure 20: Transient K_α_ XES spectra of MbNO at 0.26 (red) and 1.36 ps (blue). The 0.26 ps spectrum represents predominantly triplet state species and the 1.36 ps spectrum the quintet state. Both are normalized to the maximum of the negative signal. The error bars are the standard deviation between the runs.

# Supplementary References

1 Tono, K. *et al.* Beamline, experimental stations and photon beam diagnostics for the hard x-ray free electron laser of SACLA. *New J Phys* **15**, 083035, doi:Artn 083035, 10.1088/1367-2630/15/8/083035 (2013).

2 Ishikawa, T. *et al.* A compact X-ray free-electron laser emitting in the sub-angstrom region. *Nat Photonics* **6**, 540-544, doi:10.1038/nphoton.2012.141 (2012).

3 Silatani, M. *et al.* NO binding kinetics in myoglobin investigated by picosecond Fe K-edge absorption spectroscopy. *Proceedings of the National Academy of Sciences* **112**, 12922-12927 (2015).

4 Levantino, M. *et al.* Observing heme doming in myoglobin with femtosecond X-ray absorption spectroscopya). *Structural Dynamics* **2**, 041713, doi:doi:<http://dx.doi.org/10.1063/1.4921907> (2015).

5 Mara, M. W. *et al.* Metalloprotein entatic control of ligand-metal bonds quantified by ultrafast x-ray spectroscopy. *Science* **356**, 1276-1280 (2017).

6 Palmer, G. *et al.* Pump–probe laser system at the FXE and SPB/SFX instruments of the European X‐ray Free‐Electron Laser Facility. *Journal of synchrotron radiation* **26**, 328-332 (2019).

7 Sinn, H. *et al.* The SASE1 X-ray beam transport system. *Journal of synchrotron radiation* **26** (2019).

8 Grünert, J. *et al.* X-ray photon diagnostics at the European XFEL. *Journal of synchrotron radiation* **26** (2019).

9 Klamt, A. & Jones, V. COSMO. *J. Chem. Phys.* **105**, 9972 (1996).

10 Klamt, A. & Schüürmann, G. COSMO: a new approach to dielectric screening in solvents with explicit expressions for the screening energy and its gradient. *J. Chem. Soc., Perkin Trans. 2*, 799 - 805 (1993).

11 Klamt, A. Conductor-like screening model for real solvents: a new approach to the quantitative calculation of solvation phenomena. *The Journal of Physical Chemistry* **99**, 2224-2235 (1995).

12 Neese, F. The ORCA program system. *Wires Comput Mol Sci* **2**, 73-78, doi:Doi 10.1002/Wcms.81 (2012).

13 de Groot, F. Multiplet effects in X-ray spectroscopy. *Coordin Chem Rev* **249**, 31-63, doi:DOI 10.1016/j.ccr.2004.03.018 (2005).

14 Katayama, T. *et al.* Femtosecond x-ray absorption spectroscopy with hard x-ray free electron laser. *Applied Physics Letters* **103**, 131105 doi:Artn 131105, 10.1063/1.4821108 (2013).

15 Katayama, T. *et al.* A beam branching method for timing and spectral characterization of hard X-ray free-electron lasers. *Structural Dynamics* **3**, 034301, doi:<http://dx.doi.org/10.1063/1.4939655> (2016).

16 Milne, C. J., Penfold, T. J. & Chergui, M. Recent experimental and theoretical developments in time-resolved X-ray spectroscopies. *Coordin Chem Rev* **277**, 44-68 (2014).

17 van Stokkum, I. H. M., Larsen, D. S. & van Grondelle, R. Global and target analysis of time-resolved spectra. *Bba-Bioenergetics* **1657**, 82-104 (2004).

18 Zhang, W. K. *et al.* Tracking excited-state charge and spin dynamics in iron coordination complexes. *Nature* **509**, 345-+, doi:Doi 10.1038/Nature13252 (2014).

19 Ye, X., Demidov, A. & Champion, P. M. Measurements of the photodissociation quantum yields of MbNO and MbO(2) and the vibrational relaxation of the six-coordinate heme species. *Journal of the American Chemical Society* **124**, 5914-5924 (2002).

20 Bräm, O., Cannizzo, A. & Chergui, M. Ultrafast Broadband Fluorescence Up-conversion Study of the Electronic Relaxation of Metalloporphyrins. *The Journal of Physical Chemistry A* **123**, 1461-1468, doi:10.1021/acs.jpca.9b00007 (2019).

21 Schuth, N. *et al.* Effective intermediate-spin iron in O2-transporting heme proteins. *Proceedings of the National Academy of Sciences* **114**, 8556-8561 (2017).
